# Dietary supplementation with flaxseed meal and oat hulls modulates intestinal histomorphometric characteristics, digesta- and mucosa-associated microbiota in pigs

**DOI:** 10.1038/s41598-018-24043-5

**Published:** 2018-04-12

**Authors:** S. P. Ndou, H. M. Tun, E. Kiarie, M. C. Walsh, E. Khafipour, C. M. Nyachoti

**Affiliations:** 1 Department of Animal Science, University of Manitoba, Winnipeg, MB Canada R3T 2N2; 2 Department of Animal Biosciences, University of Guelph, Guelph, ON Canada N1G 2W1; 3 Department of Medical Microbiology, University of Manitoba, Winnipeg, MB Canada R3T 2N2; 4 DuPont Industrial Biosciences-Danisco Animal Nutrition, Marlborough, Wiltshire United Kingdom SN8 1XN

## Abstract

The establishment of a healthy gastrointestinal milieu may not only offer an opportunity to reduce swine production costs but could also open the way for a lifetime of human health improvement. This study investigates the effects of feeding soluble fibre from flaxseed meal-containing diet (FM) and insoluble fibre from oat hulls-containing diet (OH) on histomorphological characteristics, digesta- and mucosa-associated microbiota and their associations with metabolites in pig intestines. In comparison with the control (CON) and OH diets, the consumption of FM increased (*P* < 0.001) the jejunal villi height (VH) and the ratio of VH to crypt depths. The PERMANOVA analyses showed distinct (*P* < 0.05) microbial communities in ileal digesta and mucosa, and caecal mucosa in CON and FM-diets fed pigs compared to the OH diet-fed pigs. The predicted functional metagenomes indicated that amino acids and butanoate metabolism, lysine degradation, bile acids biosynthesis, and apoptosis were selectively enhanced at more than 2.2 log-folds in intestinal microbiota of pigs fed the FM diet. Taken together, flaxseed meal and oat hulls supplementation in growing pigs’ diets altered the gastrointestinal development, as well as the composition and function of microbial communities, depending on the intestinal segment and physicochemical property of the dietary fibre source.

## Introduction

Supplementing swine diets with agricultural and industrial co-products increases the dietary fibre (DF) content, which can alter the gastrointestinal (GI) milieu. The changes induced by DF include alteration of gut microbiome, enhancement of gut mucosal barrier integrity and function, increased host mucosal immunity and function, increased short chain fatty acids (SCFA) production and associated reduction in mucosal interaction of opportunistic enteric pathogens^1,2^. The SCFA produced from DF fermentation act as energy sources to the host, regulate epithelial, immune cell, and microbial growth and apoptosis^1^. Other studies have revealed that intestinal microbiota play a central role during biotransformation of primary bile acids (BA) to secondary BA and alter BA composition^3–5^. There is also accumulating evidence that BA facilitates a cross-talk between intestinal microbiota and energy or lipid metabolism^1,3,4^. Interestingly, a growing body of research has also pinpointed that BA, in turn, alter the community structure of GI microbiota^4,5^. It is intriguing to note that even when pigs are fed nutritionally balanced diets that are supplemented with fibrous ingredients and supplemental fat, growth performance is depressed^6–8^. This has stimulated further interests in understanding the relationships between SCFA and BA flows, dietary fat absorption, lipid metabolism-related blood metabolites and gut microbiota composition and function, but there is limited research to determine the effects of different DF sources and/or types.

Flaxseed meal and oat hulls are agricultural co-products that can be incorporated into diets of pigs. Flaxseeds (*Linum usitatissimum*) are rich sources of soluble non-starch polysaccharides (NSP), α-linolenic acid (ALA), and lignans^9,10^, whereas, oat (*Avena sativa*) hulls have high contents of lignin, an insoluble fibre^11,12^. Research on feeding flaxseed has primarily focused on fatty acids utilization in finishing pigs, as well as growth performance, nutrient utilization^8,13^ and ileal microbiota in weaned pigs^14^. Studies with oat hulls have also limited their focus on growth performance and nutrient digestibility in piglets^15,16^ and its ability to reduce microbial proteolysis in the large intestine^11^. In humans and rodents, both flaxseed and oat hulls have been speculatively linked with the ability to ameliorate metabolic disorders but the mechanism of action is not clear^17^. To our knowledge, very few, if any studies have been conducted to investigate the ability of flaxseed meal and oat hulls to modulate gut microbiota composition and function, intestinal morphology and functions, and their associations with GI metabolites.

Thus, the aim of the present study was to determine the effects of soluble fibre from flaxseed meal and insoluble fibre from oat hulls on histomorphological characteristics of ileal mucosa and microbiota composition and their association with gastrointestinal and blood lipid-related metabolites in the ileal- and caecal-digesta and mucosa of growing pigs fed corn and soybean meal-based diet. A bioinformatic approach (PICRUSt) was used to predict the functional properties of ileal and caecal digesta- and mucosa-associated microbial communities.

## Results

### Experimental diets

Dietary neutral detergent fibre (NDF) content (% as fed) was 9.4, 18.0 and 18.7% in the CON, FM and OH diets, respectively (Supplementary Table S2). The NSP solubility in the three treatment diets was 8.4, 26.3 and 11.9% for CON, FM and OH diets, respectively. The SWC in CON, FM, and OH diets were 2.67, 2.85 and 2.67 mL/g, respectively (Supplementary Table S2). The WHC was 3.31 mL/g in CON diet, 5.98 mL/g in FM diet, and 4.52 mL/g in OH diet.

### Intake of dietary components and growth performance measurements

The average daily gross energy (GE) intake was highest in the OH diet-fed pigs compared to pigs fed CON and FM diets (*P* < 0.001) (Supplementary Table S3). Protein intake was not significantly influenced by the treatments but metabolizable energy intake was lower in pigs supplemented FM diet (*P* = 0.006) compared to those fed CON and OH diets. Pigs that consumed the CON and FM diets had lower intake of fat (*P* < 0.001) and insoluble NSP (*P* = 0.003) than OH diets-fed pigs. The intake of soluble fibre was greatest in FM diet-fed pigs compared to the OH and CON diets-fed pigs (*P* < 0.001). However, the intake of total NSP was greater in OH diet-fed pigs compared to the FM and CON diets-fed pigs (*P* < 0.001). The SFI and FCR were not influenced (*P* > 0.05) by dietary treatment, but pigs that consumed the CON and OH diets tended (*P* = 0.060) to have a higher SADG compared to those fed the FM diet.

### Dietary inclusion of flaxseed meal and oat hulls modulates intestinal histomorphometric characteristics

Addition of flaxseed meal and oat hulls in pig diets decreased (*P* < 0.001) the villi height (VH) in the duodenum and ileum compared to CON diet (Supplementary Table S4). The jejunal VH were higher (*P* < 0.001) in FM diet-fed pigs compared to CON and OH diets-fed pigs. Although there was a tendency in which crypt in duodenum of pigs fed FM diets was deeper (*P* = 0.057) compared to those fed OH diets, no significant differences were observed in jejunal and ileal crypt depth (CD) among treatments. Dietary inclusion of flaxseed meal decreased (*P* < 0.037) the ileal VH:CD ratio and tended to decrease (*P* = 0.073) the duodenal VH:CD ratio, but increased (*P* < 0.001) jejunal VH:CD in comparison to CON and OH diets.

### Supplementation with flaxseed meal and oat hulls alters gastrointestinal tract (GIT) environment

#### Alpha-diversity differences in the ileal and caecal microbial communities

As illustrated in Supplementary Table S5 and Fig. S1, no significant diet-induced effects were observed on Chao1 richness as well as Shannon and Simpson diversity indices in ileal digesta microbiota. Although there were no significant differences observed on Chao1 richness (*P* = 0.828) and Simpson diversity (*P* = 0.365) indices in ileal mucosa-associated microbiota, there was a tendency (*P* = 0.064) in which the greatest Shannon diversity value was observed in FM diet-fed pigs, followed by OH and CON-diet fed pigs (Supplementary Table S6 and Fig. S2). The Shannon diversity index indicated no treatment effects (*P* > 0.10) within caecal digesta microbiota but the Chao1 index revealed a tendency in which species richness in CON diet-fed pigs was lower (*P* = 0.05) compared to pigs consumed OH and FM diets (Supplementary Table S5 and Fig. S3). According to Shannon diversity index calculated in caecal mucosa, higher (*P* = 0.015) bacterial diversity was observed in both FM and CON diets-fed pigs compared to OH (Supplementary Table S6 and Fig. S4). The comparison of Chao1 index within caecal mucosa revealed that species richness was higher (*P* = 0.039) in FM diet-fed pigs compared to those fed the CON diet. There were no treatment effects on Simpson diversity index in caecal mucosa-associated microbiota (*P* = 0.144).

### Assessment of beta-diversity differences in ileal and caecal microbiota

**Figure 1:**
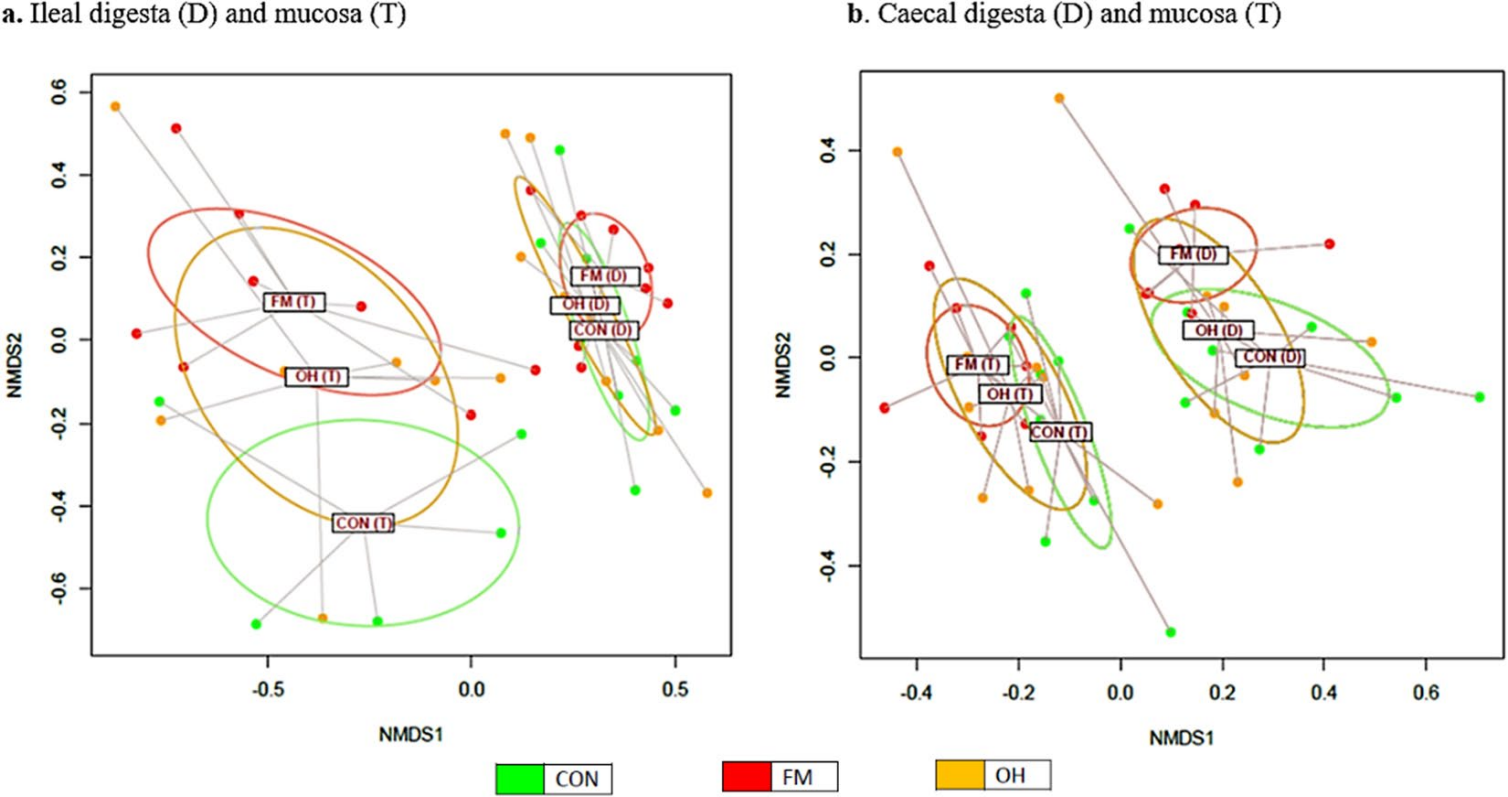
Non-metric multidimensional scaling (nMDS) ordination plot, illustrating beta biodiversity differences of bacterial community compositions in the caecal digesta (D) and mucosa-associated microbiota (T) of pigs fed control (CON), flaxseed meal (FM) and oat hulls (OH) diets. The PERMANOVA analyses of unweighted UniFrac distances revealed distinct clustering patterns between the ileal digesta microbiota of CON and FM (*P* = 0.010), and FM and OH diets-fed pigs (*P* = 0.005). The PERMANOVA analysis of unweighted UniFrac distances also revealed distinctions between caecal mucosa-associated microbiota of the CON and FM-diets fed pigs (*P* = 0.030), and ileal mucosa-associated microbiota of the FM and OH-diets fed pigs (*P* = 0.041).

The assessment of β-diversity differences in ileal and caecal microbiota are illustrated in Fig. 1a,b. The PERMANOVA analyses of unweighted UniFrac distances revealed distinct clustering patterns between the ileal digesta microbiota of CON and FM (*P* = 0.010), and FM and OH diets-fed pigs (*P* = 0.005). The PERMANOVA analysis of unweighted UniFrac distances also revealed distinctions between caecal mucosa-associated microbiota of the CON and FM-diets fed pigs (*P* = 0.030), and ileal mucosa-associated microbiota of pig fed FM and OH diets (*P* = 0.041).

### Composition of ileal digesta and mucosa-associated microbiota

**Figure 2:**
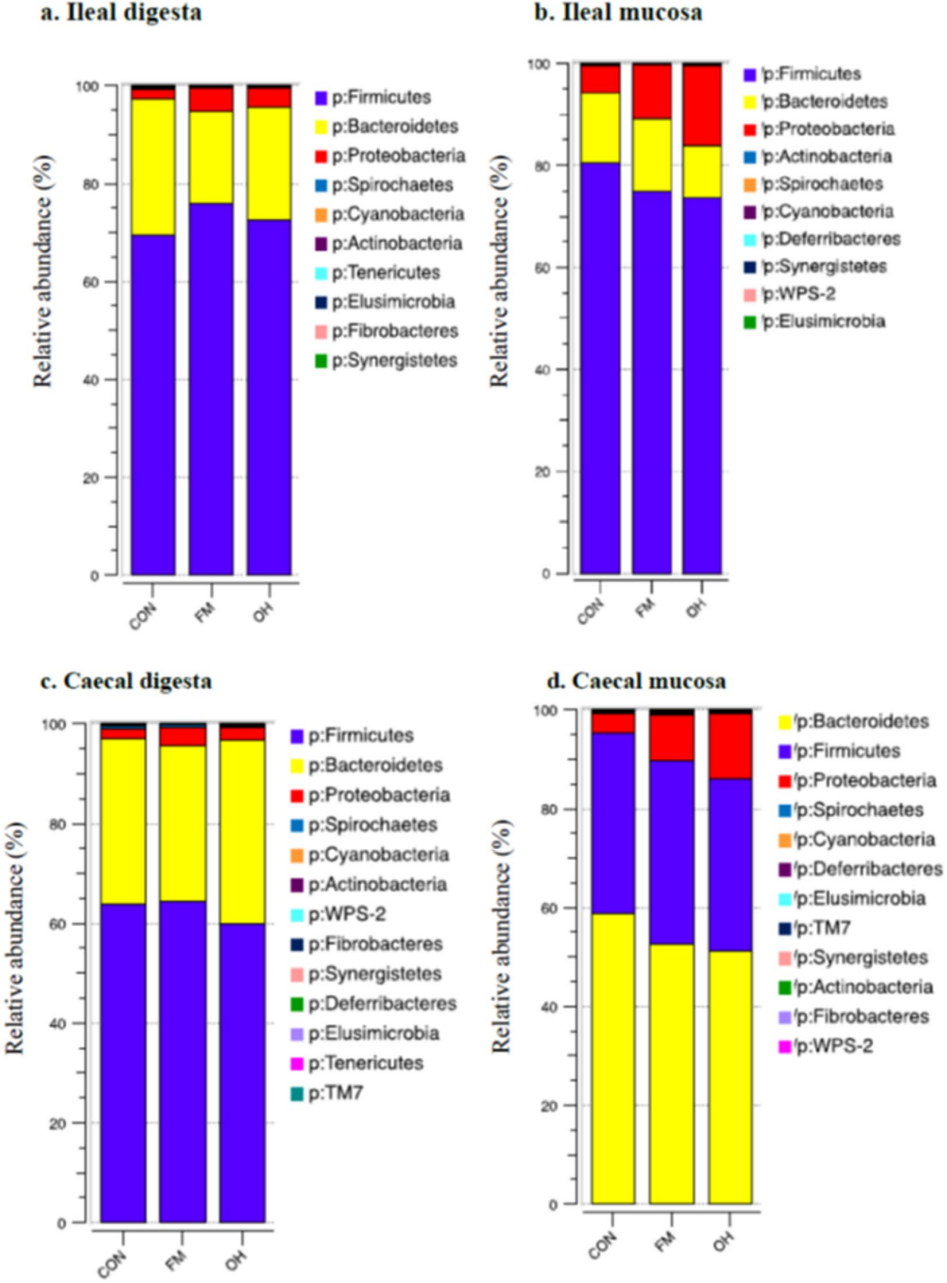
Relative abundances of bacterial phyla (p) in the: (**a**) ileal digesta; (**b**) ileal mucosa (**c**); caecal digesta (**d**); and caecal mucosa-associated microbiota of pigs fed the control (CON); flaxseed meal (FM) and oat hulls (OH) diets. The statistical differences were calculated using GLIMMIX procedure and were presented in Supplementry Tables S7, S8, S11 and S12.

**Figure 3:**
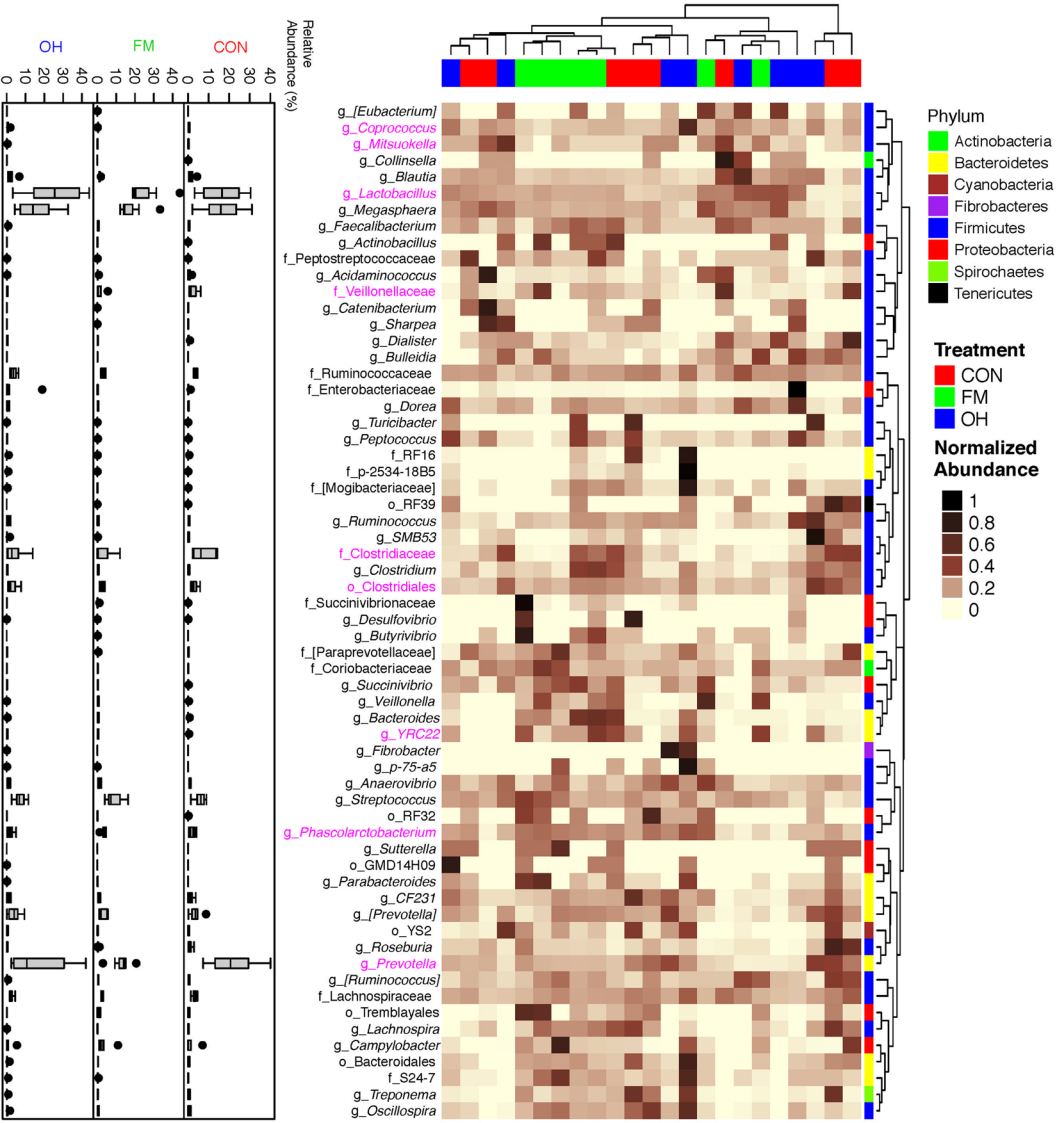
Cluster analysis of microbial communities in the ileal digesta of pigs fed control diet (CON), flaxseed meal (FM) and oat hulls (OH) diets. The sample identifiers on the top branches for diet treatments are coloured. Right branches are coloured to indicate the taxonomical assignment of the OTUs at the phylum level. Each row represents one bacterial taxa (relative abundance above 0.01%), and some taxa could only be classified to family (f), order (o), class (c), or phylum (p) level. The taxa names were labeled by the colour code (magenta) to indicate the abundance of the taxa significantly differed between diet treatments (P < 0.05). The normalized relative abundance of bacterial taxa from illumina-sequenced 16 S rRNA sequences in each sample is reflected by the colour of the scale (light yellow to black) on the heat map. The dendrogram on the top shows how the samples are clustered based on the Bray–Curtis dissimilarity measure, averaged by diet treatment. The dendrogram on the right shows clustering of bacterial taxa data based on the Spearman’s rank correlation. The Box-Plots showed the relative abundances of bacterial taxa in ileal digesta microbiota among the three diet treatments.

**Figure 4:**
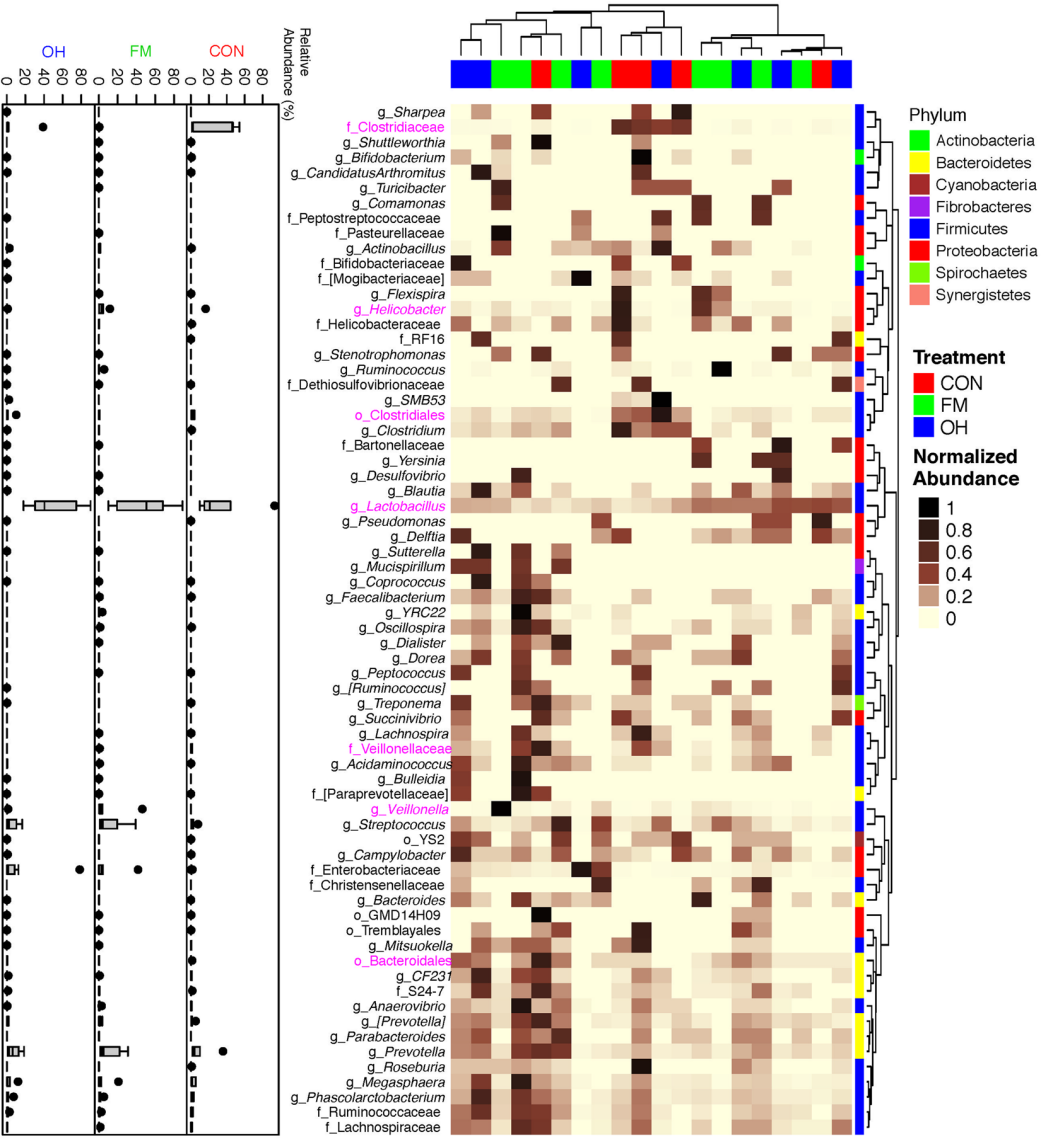
Cluster analysis of microbial communities in the ileal mucosa of pigs fed control diet (CON), flaxseed meal (FM) and oat hulls (OH) diets. The sample identifiers on the top branches for diet treatments are coloured. Right branches are coloured to indicate the taxonomical assignment of the OTUs at the phylum level. Each row represents one bacterial taxa (relative abundance above 0.01%), and some taxa could only be classified to family (f), order (o), class (c), or phylum (p) level. The taxa names were labeled by the colour code (magenta) to indicate the abundance of the taxa significantly differed between diet treatments (P < 0.05). The normalized relative abundance of bacterial taxa from illumina-sequenced 16 S rRNA sequences in each sample is reflected by the colour of the scale (light yellow to black) on the heat map. The dendrogram on the top shows how the samples are clustered based on the Bray–Curtis dissimilarity measure, averaged by diet treatment. The dendrogram on the right shows clustering of bacterial taxa data based on the Spearman’s rank correlation. The Box-Plots showed the relative abundances of bacterial taxa in ileal mucosa microbiota among the three diet treatments.

Taxonomic classification of clustered OTUs in the ileal microbiota revealed the presence of 10 bacterial phyla. While some of OTUs were identified at the genus (g.) or species levels, others were only classified at the phylum (p.), class (c.), order (o.), or family (f.) level. Firmicutes were the most abundant phylum, followed by Bacteroidetes and Proteobacteria, with pigs fed the FM and OH diets having (*P* = 0.081) the greatest proportion of p. Firmicutes in digesta compared to pigs fed CON diet (Fig. 2a, Supplementary Table S7). Ileal mucosa-associated microbiota, p. Firmicutes were most (*P* = 0.001) dominant in CON diet-fed pigs compared to those fed the FM and OH diets (Fig. 2b, Supplementary Table S8). However, p. Bacteroidetes did not differ (*P* = 0.772) among treatment groups in ileal digesta but was highest (*P* < 0.001) in ileal mucosa of FM diet-fed pigs and lowest in pigs fed the OH diet compared to those fed the CON diet. The compositions of bacterial taxa at the genus level in the ileal digesta and mucosa of pigs fed the experimental diets are presented in Supplementary Tables S9 and S10, respectively. As illustrated by differences in colour codes on the heat map, the cluster analysis of microbial community indicated that the abundance of taxa in ileal digesta (Fig. 3) and mucosa (Fig. 4) differed (*P* < 0.05) between diet treatments.

### Composition of caecal digesta and mucosa-associated microbiota

**Figure 5:**
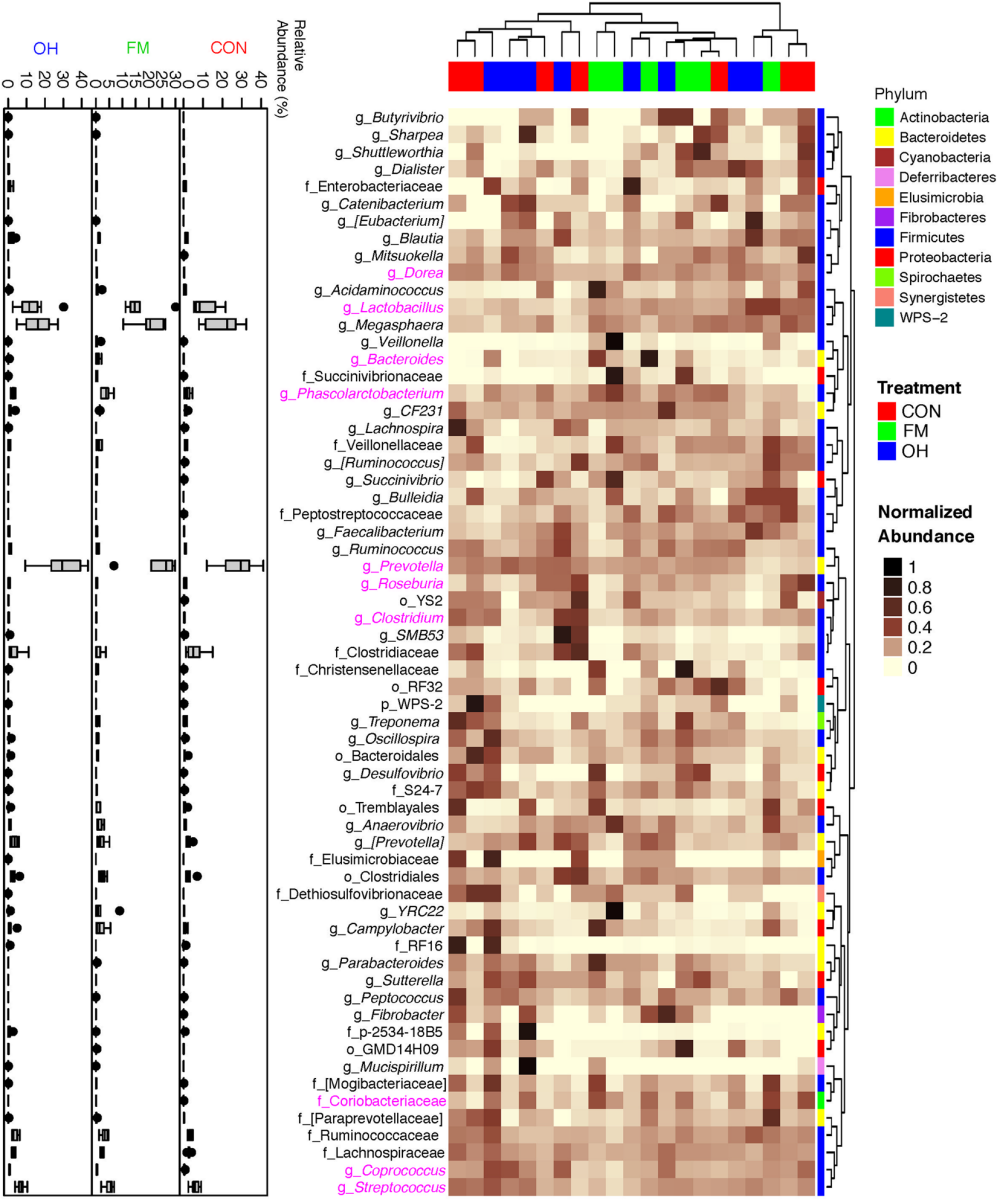
Cluster analysis of microbial communities in the caecal digesta of pigs fed control diet (CON), flaxseed meal (FM) and oat hulls (OH) diets. The sample identifiers on the top branches for diet treatments are coloured. Right branches are coloured to indicate the taxonomical assignment of the OTUs at the phylum level. Each row represents one bacterial taxa (relative abundance above 0.01%), and some taxa could only be classified to family (f), order (o), class (c), or phylum (p) level. The taxa names were labeled by the colour code (magenta) to indicate the abundance of the taxa significantly differed between diet treatments (P < 0.05). The normalized relative abundance of bacterial taxa from illumina-sequenced 16 S rRNA sequences in each sample is reflected by the colour of the scale (light yellow to black) on the heat map. The dendrogram on the top shows how the samples are clustered based on the Bray–Curtis dissimilarity measure, averaged by diet treatment. The dendrogram on the right shows clustering of bacterial taxa data based on the Spearman’s rank correlation. The Box-Plots showed the relative abundances of bacterial taxa in caecal digesta microbiota among the three diet treatments.

**Figure 6:**
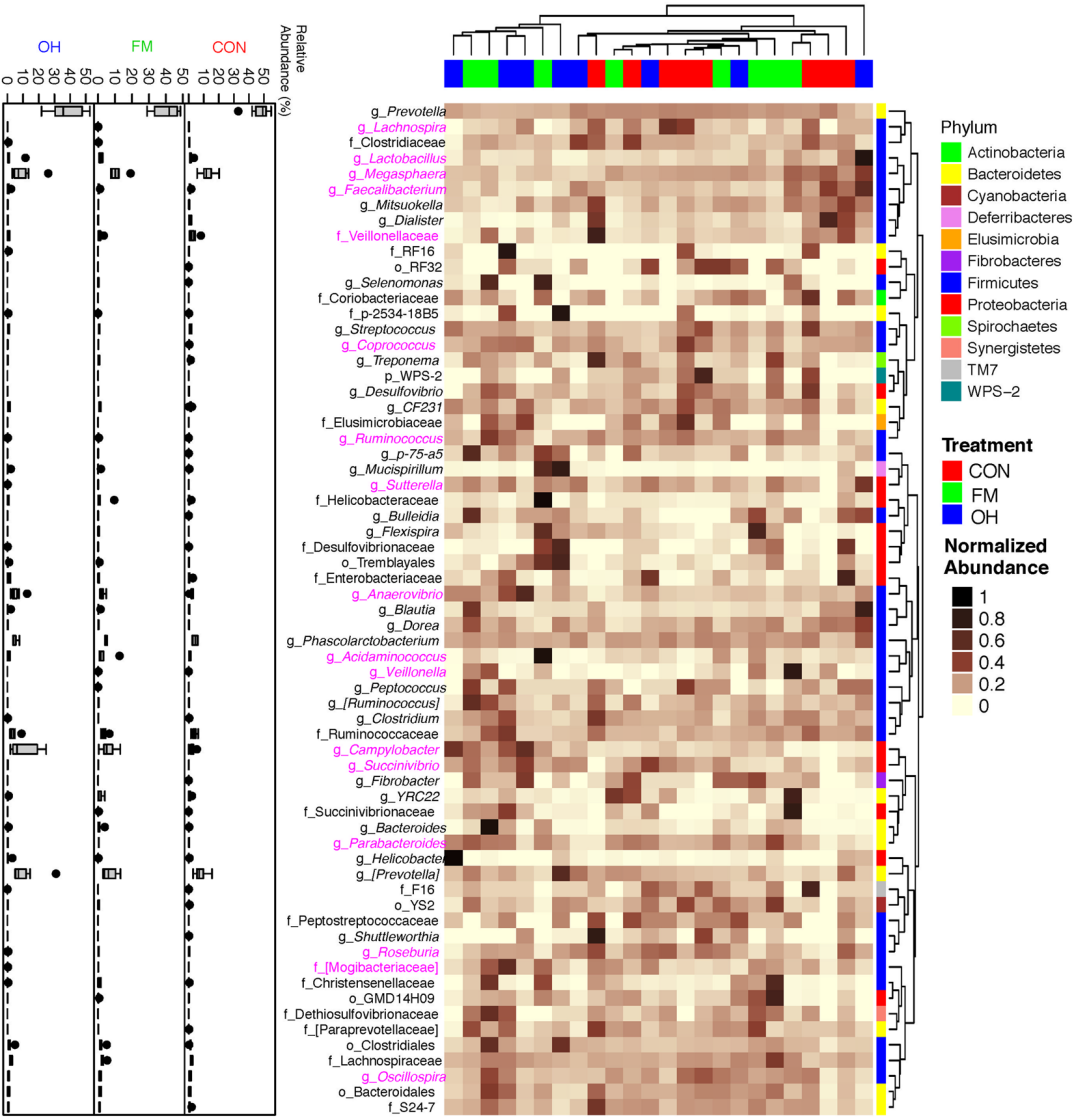
Cluster analysis of microbial communities in the caecal mucosa of pigs fed control diet (CON), flaxseed meal (FM) and oat hulls (OH) diets. The sample identifiers on the top branches for diet treatments are coloured. Right branches are coloured to indicate the taxonomical assignment of the OTUs at the phylum level. Each row represents one bacterial taxa (relative abundance above 0.01%), and some taxa could only be classified to family (f), order (o), class (c), or phylum (p) level. The taxa names were labeled by the colour code (magenta) to indicate the abundance of the taxa significantly differed between diet treatments (P < 0.05). The normalized relative abundance of bacterial taxa from illumina-sequenced 16 S rRNA sequences in each sample is reflected by the colour of the scale (light yellow to black) on the heat map. The dendrogram on the top shows how the samples are clustered based on the Bray–Curtis dissimilarity measure, averaged by diet treatment. The dendrogram on the right shows clustering of bacterial taxa data based on the Spearman’s rank correlation. The Box-Plots showed the relative abundances of bacterial taxa in caecal mucosa microbiota among the three diet treatments.

The taxonomic classification of clustered OTUs in the caecal microbiota revealed the presence of 13 bacterial phyla in digesta (Fig. 2c, Supplementary Table S11) and 12 bacterial phyla in mucosa (Fig. 2d, Supplementary Table S12). The majority of OTUs were identified at the genus (g.) or species levels, but some were only classified at the phylum (p.), class (c.), order (o.), or family (f.) level. Among the most dominant phyla in caecal digesta, the proportion of Firmicutes was higher (*P* < 0.001) in pigs fed the FM diet compared to those fed OH and CON diets (Fig. 2c, Supplementary Table S11). Although no significant differences were observed on the proportion of p. Bacteroidetes across all treatments, the proportion of p. Proteobacteria was higher (*P* = 0.049) in FM diet-fed pigs compared to that of pigs fed the OH and CON diets. As depicted in Fig. 2d and Supplementary Table S12, p. Bacteroidetes were the most dominant phyla in caecal mucosa and their proportion was lowest (*P* = 0.008) in OH diet-fed pigs compared to the CON. No significant differences were observed in the proportions of p. Firmicutes among treatments. However, the proportion of Proteobacteria in caecal mucosa of OH diet-fed pigs were higher (*P* < 0.001) compared to CON. The compositions of bacterial genera in caecal digesta and mucosa-associated microbiota of pigs fed the experimental diets are presented in Supplementary Tables S13 and S14, respectively. As shown by differences in colour codes on the heat map, the cluster analysis of microbial community indicated that the abundance of the taxa in caecal digesta (Fig. 5) and mucosa (Fig. 6) differed (*P* < 0.05) between diet treatments.

### Predicted functional capacity of the intestinal microbiota

**Figure 7:**
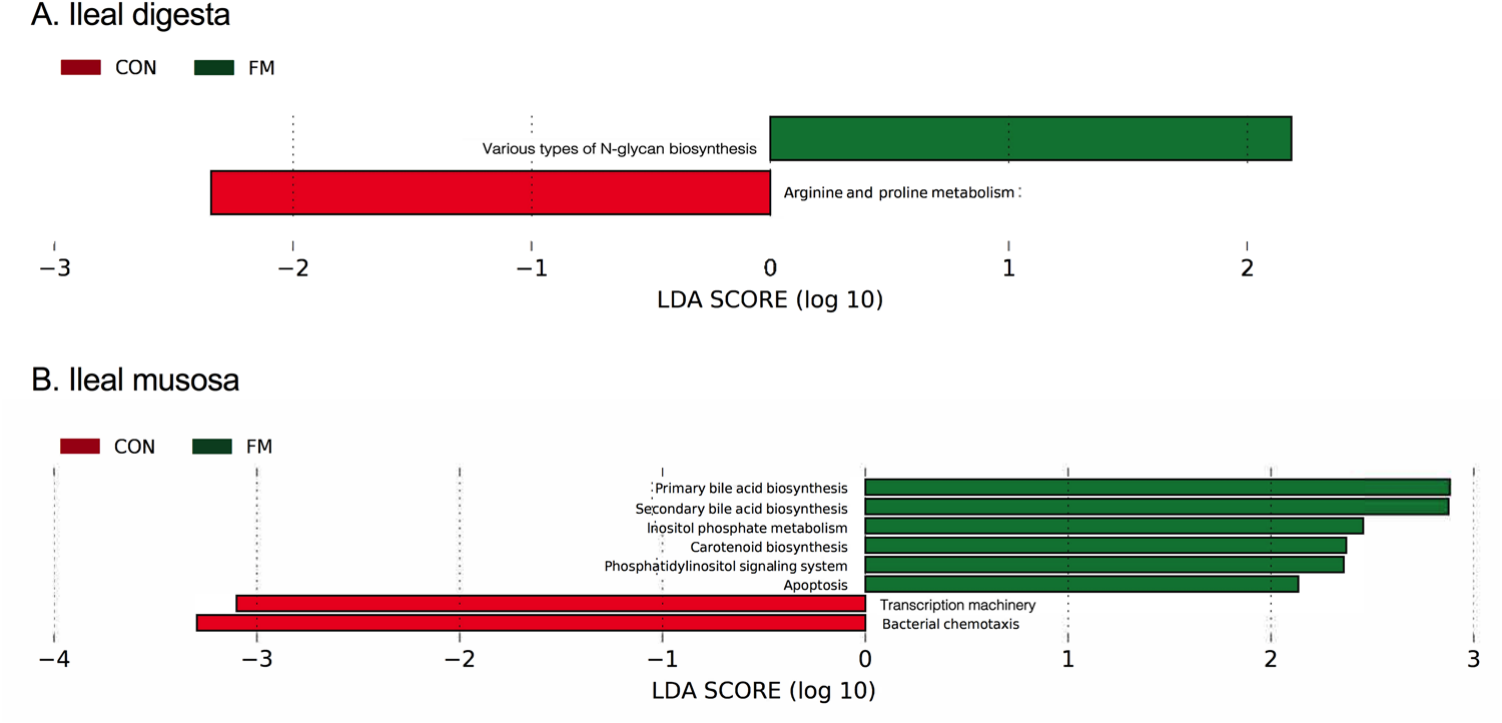
Predicted functional metagenomes of ileal digesta (**A**) and mucosa (**B**) microbiota of pigs fed control (CON) and flaxseed meal (FM) diets. Linear discriminant analysis (LDA) was performed to identify significant changes in the proportion of reconstructed functional pathways obtained from PICRUSt predictive algorithms at Kyoto Encyclopaedia of Genes and Genomes (KEGG; level 2 and 3). Analysis was performed using linear discriminant analysis of effect size (LEfSe), a metagenome analysis approach which performs the LDA following the Wilcoxon Mann-Whitney test to assess effect size of each differentially abundant variable. Colour code represents the class of treatment. Red indicates variables that were detected as significantly (Log LDA > 2.00) more abundant in CON whereas green indicates variables that were detected as significantly more abundant in FM diet-fed pigs).

As illustrated in Fig. 7A, various types of N-glycan biosynthesis mechanisms were overrepresented at more than 2.2 log-folds within ileal digesta microbiota of FM diet-fed pigs, whereas arginine and proline metabolism were enriched in ileal digesta microbiota of CON diet-fed pigs. Figure 7B depicts that functional pathways including primary BA, secondary BA and carotenoid biosynthesis, phosphatidylinositol signalling system and apoptosis were enriched in ileal mucosa-associated microbiota of FM diet-fed pigs. In addition, the Kyoto Encyclopaedia of Genes and Genomes (KEGG) functional pathways including transcription machinery and bacterial chemotaxis indicated more than 3.1 log-fold increase within the ileal mucosa of CON diet-supplemented pigs (Fig. 7B).

**Figure 8:**
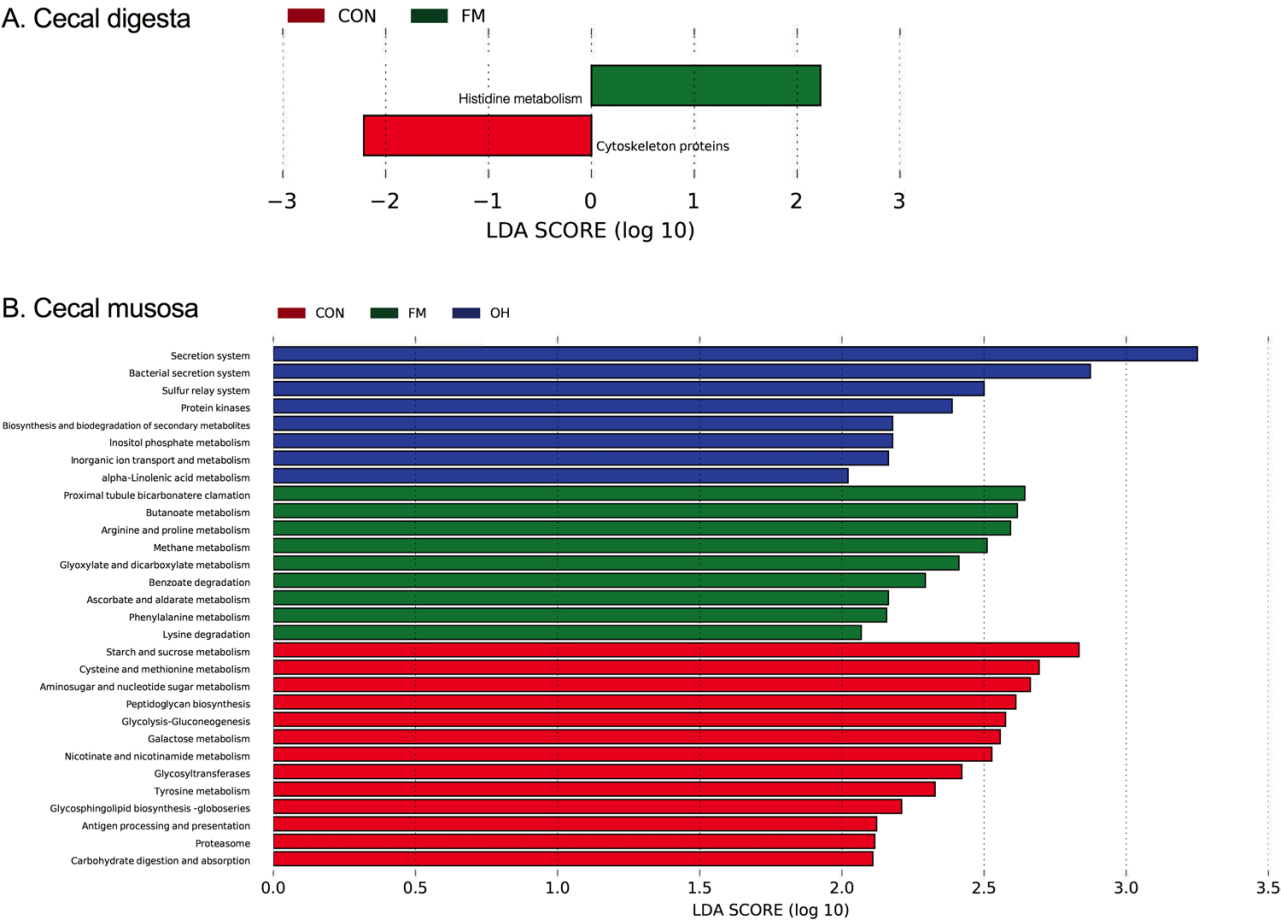
Predicted functional metagenomes of in caecal digesta (**A**) and mucosa (**B**) microbiota of pigs fed the control (CON) and flaxseed meal (FM) diets. Linear discriminant analysis (LDA) was performed to identify significant changes in the proportion of reconstructed functional pathways obtained from PICRUSt predictive algorithms at Kyoto Encyclopaedia of Genes and Genomes (KEGG; level 2 and 3). Analysis was performed using linear discriminant analysis of effect size (LEfSe), a metagenome analysis approach which performs the LDA following the Wilcoxon Mann-Whitney test to assess effect size of each differentially abundant variable. Colour code represents the class of treatment. Red indicates variables that were detected as significantly (Log LDA > 2.00) more abundant in CON, whereas green indicates variables that were detected as significantly more abundant in FM diet-fed pigs. Blue indicates variables that were detected as significantly more abundant in OH-diet fed pigs.

Functional pathways enriched within caecal digesta microbiota in FM diet-fed pigs were histidine metabolism, whereas cytoskeleton proteins were augmented in caecal digesta microbiota in CON diet-fed pigs (Fig. 8A). As shown in Fig. 8B, various functional pathways were enriched by more than 2.0 log-fold increase within ileal mucosa-associated microbiota across all treatments. Specifically, KEGG pathways including secretion, bacterial secretion and sulphur relay systems, protein kinases and biosynthesis and biodegradation of secondary metabolites, inorganic ion transport and metabolism of inositol phosphate, alpha-linoleic acid, and inorganic ion were enriched in OH diet-fed pigs. Furthermore, functional pathways including proximal tubule bicarbonate reclamation, benzoate and lysine degradation, as well as butanoate, arginine and proline, glyoxylate and dicarboxylate, ascorbate and aldarate, and phenylalanine metabolism were enriched in FM diet-fed pigs. Moreover, the most significantly enriched functional pathways in ileal mucosa of CON-fed pigs were starch and sucrose, cysteine and methionine, amino sugar and nucleotides sugar, galactose, nicotinate and nicotinamide, and tyrosine metabolism; peptidoglycan and glycosphingolipids biosynthesis, as well as glycolysis-gluconeogenesis, glycosyltransferases, antigen processing and presentation, proteasome, and carbohydrate digestion and absorption.

### Potential connections between specific intestinal digesta and mucosa-associated microbiota, gut metabolites and blood lipids

Significant correlations were observed between selected taxa, with GI metabolites and lipid metabolism-related blood metabolites (Supplementary Tables S15–18). In ileal digesta, positive correlations were observed between g. *Lactobacillus* and CA-CDCA (Rho = 0.427; *P* = 0.023), g. *Streptococcus* and fat digestibility (Rho = −0.417; *P* = 0.017). The presence of g. *Lactobacillus* was negatively correlated with IDCA (Rho = −0.644; *P* < 0.001), whereas f. Veillonellaceae was positively correlated acetate (Rho = 0.534; *P* = 0.002). In ileal mucosa, g. *Lactobacillus* is positively correlated with propionate, LCA, DCA and IDCA; whereas g. *Veillonella* spp. is positively correlated with propionate, butyrate and valerate (*P* < 0.05). In caecal digesta, g. *Clostridium*, g. *Prevotella*, g. *Coprococcus*, and g. *Dorea* were negatively correlated with butyrate, but f. Coriobacteriaceae and g. *Acidiminococcus* were positively correlated with butyrate (*P* < 0.05).

## Discussion

The cross-talk between diet, gut microbiota, host’s GI tract epithelium, GI metabolites and overlying mucus layer is complex and partly relies on amount and type of DF. Our recent findings showed that flaxseed meal and oat hulls supplementation in growing pigs’ diets induced variable effects on growth performance, blood lipids, intestinal fermentation, bile acids, and neutral sterols^8^. The present study is the first to compare the effects of feeding flaxseed meal and oat hulls on intestinal histomorphological features and digesta and mucosa-associated microbiota. As mentioned above, gut microbiota can modulate the pool and composition of both BA and SCFA, and in turn, these GI metabolites alter the richness and diversity of communal structure of intestinal microbiome^3,4^, but there is limited research about effects of practical sources of DF fed to farmed animals.

Histological attributes of the intestines are modulated by DF, but their growth and development was critically associated with optimal growth performance in pigs^18,19^. For example, a rise in butyrate and nutrient concentration in the intestinal lumen increases VH or deeper crypts^20,21^, and is associated with an increased absorptive capacity of intestines and a healthy gut^22^. In the present study, an increase in jejunal VH was observed in pigs which consumed the FM diet compared to those fed the CON and OH diets, suggesting an increase of ingesta in the lumen^20,21,23^.

Interestingly, the microbial metagenomics activities in the current study indicated that the KEGG functional pathways of apoptosis were elevated in ileal mucosa of pigs fed the FM diet and occurred at the same time with a decrease in the ileal VH and VH:CD ratio in pigs fed the FM-diet. Apoptosis can be defined as a mechanism where excess or redundant cells are degenerated and removed during development and restricted tissue size is maintained as a normal and controlled process of an organism’s growth or development^24^. The increase in the apoptosis could have been a compensatory mechanism to open the way for replenishing mucosal cells following the damage which may be indirectly caused by the viscous characteristics of the NSP mucilage in flaxseed meal^25^. Butyrate had been shown to increase apoptosis in human colonic cell lines^24^. Reinforcing this idea is the observation in the present study that the KEGG metagenomics pathways of butyrate metabolism in ileal mucosa-associated microbiota were increased by more than 2.5 fold in pigs fed the FM diet. Moreover, there was a positive correlation between butyrate concentration and *Veillonella* spp., a well-known lactate-fermenting bacterium^26^.

A similar trend was observed on the composition of bacterial communities in ileal digesta from both FM and OH diets-fed pigs where members of the Firmicutes phyla were the most dominant followed by smaller relative abundance of Bacteroidetes and Proteobacteria. The findings demonstrated that an increase in DF intake induced a shift in microbial composition due to the presence of substrate that promote growth of beneficial phyla and reduce the abundance of BA-tolerant species in ileal digesta^27,28^. Previous studies have also revealed that BA in intestinal digesta suppresses growth of p. Bacteroidetes and Actinobacteria, and consequently exert beneficial effects on p. Firmicutes, especially BA-7α-dehydroxylating species^4^. Supporting these notions are positive correlations between bacterial order Clostridiales and family Clostridiaceae with secondary BA (lithocholic acid (LCA), deoxycholic acid (DCA), isodeoxycholic acid (IDCA), ursodeoxycholic acid (UDCA) observed in the current study supports the notion that g. *Clostridium* are the only members of the Firmicutes phylum that possess 7α/β-dehydroxylase and catalyze the hydrogenation reaction of primary BA to generate secondary BA^29–31^. Furthermore, a positive correlation between g. *Lactobacillus* and primary acids (CD-CDAC) and secondary IDCA observed in the current study agrees with the notion by Begley *et al*.^32^, and categorically indicates that gut commensal phyla tolerate BA by expressing bile salt hydrolase (BSH). The observations that order Clostridiales and Clostridiaceae families in ileal digesta, and g. *Clostridium* and Clostridiaceae families in caecal digesta were positively correlated with secondary BA, such as LCA, DCA, IDCA and UDCA are also consistent with reports by Ridlon *et al*.^33,34^. *Clostridium* and *Eubacterium* genera are members of Firmicutes phylum that convert primary BA into secondary forms through gut microbial 7-dehydroxylation and 7 α/β-epimerization^5^. The observation that the Firmicutes phyla were the most dominant in ileal mucosa of pigs fed the control diet compared to that of pigs which consumed FM and OH diets was unexpected and is difficult to explain with variables measure in the current study. However, the positive correlation observed between g. *Streptococcus* and DCA in ileal mucosa is supported by Salvioli *et al*.^35^ who reported that administration of *Streptococcus* spp, bacteria that produce substances acting against c. Clostridia reduced cholesterol saturation and molar percentage of DCA in bile and consequently increased CA and DCA in faeces. Moreover, flaxseed meal and oat hulls supplementation reduced the absorptive capacity of intestinal mucosa by decreasing VH, and indirectly promoted BA deconjugation^36–38^. Metagenomic analyses in previous studies have revealed that functional BSH activities are present in all major bacterial divisions in human gut including members of *Lactobacilli*, *Bifidobacteria*, *Clostridium* and *Bacteroides*^33,36,37,39^. Interestingly, the microbial metagenomic analysis in the current study indicated that KEGG functional pathways of primary BA and secondary BA biosynthesis were enriched in ileal mucosal microbiota of FM diet-fed pigs. Thus, ingestion of flaxseed meal fibre could promote BA biotransformation by altering intestinal microbiota in a beneficial fashion in human nutrition. Conversely, this could have adverse effects in swine nutrition because BA formation and enterohepatic circulation (EHC) occurs at the expense of cholesterol and metabolizable energy needed for growth performance. In fact, EHC occurs in humans about six times a day^5^ and BSH is enriched in gut microbiota compared to other microbial ecosystem^39^. In this regard, EHC and microbial species should be investigated in more detail to understand why performance is depressed in pigs that are fed nutritionally balanced high-fibre diets that are also supplemented with vegetable oil.

Although a statistical difference was not observed for g. *Clostridium* among treatments, p. Firmicutes members in ileal digesta; *Lactobacillus*, Clostridiales and Clostridiaceae increased in abundance in pigs fed the FM and OH diets, whereas, g. *Prevotella* were negatively influenced by intake of FM diets only. The relative increase in abundance of g. *Lactobacillus* is supported by their positive correlation with ileal propionate in the present study and by findings from previous studies performed *in vivo* using weaned pigs^14^ and adult men^17^, and *in vitro* using cow gut microbiota^40^. These studies demonstrated that fermentation of flaxseed fibres yields a remarkably high proportion of propionic acid. Because g. *Lactobacillus* increase was associated with a lower abundance of g. *Faecalibacterium* also reported by Berggren *et al*.^41^, it can be speculated that the mechanism by which the former suppresses the growth of the latter species is by competitive exclusion as well changes in enzymatic activities^42^. Lactobacilli and streptococci are major species in the pig intestine and can convert carbohydrates into lactic acid^43^. The cross-feeding theory suggests that lactic acid can be utilized by *Veillonella* spp as a carbon source and converted into propionate and acetate^26^. Thus, findings in the present study that respective increases in lactobacilli and streptococci were associated with increased Veillonellaceae families in ileal digesta and *Veillonella* spp in caecal mucosa of pigs that consumed flaxseed meal supports this phenomenon. Furthermore, the strong correlations observed between Veillonellaceae families and propionate in ileal digesta; g. *Veillonella* and propionate in ileal mucosa; and f. Veillonellaceae with acetate in caecal mucosa are further indicators of the presence of bacteria that do not ferment fibre but use fermentation products that are produced by others in the gut^26^. Thus, the increased g. *Lactobacillus* in FM diet fed pigs supports the suggestions by Kiarie *et al*.^14^ that there are great opportunities for using flaxseed meal to modify intestinal microbial activity in a beneficial fashion.

Although the proportion of other p. Firmicutes such as o. Clostridiales and f. Clostridiaceae increased with intake of FM in the ileum, these microbial communities were underpopulated in the ileal mucosa, caecal digesta and caecal mucosa, compared to the CON and OH diets-fed pigs. The suppression of these beneficial members is difficult to explain but could be attributed to the fact that during digesta transit from ileum to cecum there is a concomitant reduction in the quantities of substrate needed to support their microbial fermentation. However, positive correlations observed between Clostridiaceae families in cecum digesta and g. *Lachnospira* in caecal mucosa with butyrate concentrations in this study are in agreement with the common dogmatic belief that these microbial communities generate butyrate^44^. There is a need for future studies to evaluate DF degradation in different segments of the gut.

Protein that escapes digestion in the upper gut can be fermented by microbial communities in the hindgut to produce branched-chain amino acids^2^. The proportions of p. Proteobacteria in caecal digesta were highest in FM, whereas p. Bacteroidetes colonized caecal mucosa of OH diets-fed pigs. Bacteroidetes and Proteobacteria phyla and in particular p. Bacteroidetes have been linked with amino acid metabolism to produce branched-chain VFA (BCVFA), such as isovalerate and isobutyrate^45^. Although it is well-known that the host cannot metabolize bacterial protein or BCVFA^45^, it is interesting to note that in the current study functional pathways enriched with microbial activity in caecal digesta of FM diets-fed pigs included histidine metabolism. Moreover, functional pathways enriched with microbial activity in caecal mucosa of FM-fed pigs included arginine, proline, and phenylalanine metabolism and those enriched in OH diets-fed pigs included cysteine, methionine and tyrosine metabolism. In general, these observations suggest that deposition of the amount or composition of proteins that resist digestion in the upper gut is influenced by differences in the physicochemical properties of the DF sources. For example, due to its high WHC and SWC measured in this trial and its ability to increase digesta viscosity reported by Kiarie *et al*.^14^, flaxseed meal could depress protein digestibility thereby increasing the amino acid pool available for hindgut degradation. Supporting this postulation is the observation in the present study that lysine degradation is another functional pathway that was enriched in caecal mucosa-associated microbiota of pigs that consumed FM diets. A recently published study of an extensive metagenomics approach has revealed that lysine can also be incorporated into bacterial biosynthetic pathway to produce butyrate^46^. Conversely, oat hulls are classified as sources of insoluble DF and they could have depressed ileal amino acid digestibility by increasing digesta passage rate thereby increasing amount of amino acids that are available for hindgut microbial activity. The use of ileal-cannulated pigs would open way for future studies to test these postulations.

In conclusion, addition of flaxseed meal and oat hulls in corn-soybean meal-based diets alters the histological attributes of the small intestines. Flaxseed meal and oat hulls supplementation also induced variable effects on microbial communities at both phylum and lower taxonomic levels, depending on intestinal segment and physicochemical property of the fibrous ingredients. The intake of either soluble fibre from flaxseed meal or insoluble fibre from oat hulls modulates associations between microbiota and metabolites as well as their predicted metagenomic functions. More work is needed to estimate the fatty acids flows in each gut segment and also identify the actual microbial species within each genera in pigs fed diets enriched with either flaxseed meal or oat hulls.

## Materials and Methods

### Ethical considerations

The experimental procedures for this study were approved by the University of Manitoba Animal Care (Protocol Number: F13-027) and pigs were cared for in accordance with the guidelines of the Canadian Council on Animal Care^47^.

### Animals and Housing

A total of 48 Genesus [(Yorkshire-Landrace♀) × Duroc ♂] barrows with an initial mean body weight (BW) of 25 ± 0.32 kg (mean ± SEM) were obtained from University of Manitoba’s Glenlea Swine Research Unit (Winnipeg, MB, Canada). The pigs were housed in pairs and randomly allocated to the experimental diets for 30 d. Therefore, there were 8 replicate pens in each treatment with 2 pigs per pen. Feed and water were provided at all times throughout the experiment. Weekly measurements of feed intake and BW were conducted from d 0 to d 28 to determine scaled feed intake (SFI), scaled weight gain (SADG) and feed conversion ratio (FCR). The temperature in the experimental room was maintained at 21.2 ± 2.3 (mean ± SD) °C.

### Dietary fibre sources and experimental diets

Flaxseed meal, a co-product from the mechanical pressing of flaxseed to produce flax oil, was sourced from Shape Foods Inc. (Brandon, MB, Canada). Oat hulls, a co-product from the mechanical extraction of groats (edible huskless grains) from oat kernels, were supplied by Grain Millers Inc. (Yorkton, SK, Canada). Flaxseed meal and oat hulls were selected based on the differences in their DF composition and solubility, protein content, ether extract, water holding capacity (WHC), bulk density (BD) and swelling capacity (SWC) (Supplementary Table S1). The assumption behind the selection was that during transit in the gut, the two fibrous ingredients would exclusively induce unique fermentation kinetics, SCFA-profiles, lipid metabolism as well as differential effects on the composition of lower gut microbiota.

Three iso-energetic diets were formulated to contain similar standardized ileal digestible (SID) contents, and meet other nutrient requirements for growing pigs between 25 and 50 kg BW (NRC, 2012). The diets were based on corn and soybean meal-containing; 0% flaxseed meal or oat hulls (control, CON), a 12% flaxseed meal (FM), and 10% oat hulls (OH) (Supplementary Table S1). The CON was designed to be a basal diet with DF content typical of a commercial diet. Titanium dioxide (TiO_2_) was included in all diets to determine the apparent total tract digestibility (ATTD) of fat.

### Analyses of experimental diets and sampling of intestinal tissue and digesta

The samples of experimental diets were ground through a 2 mm screen and analysed in duplicate for their chemical and physical properties (Supplementary Table S2) following procedures described by Ndou *et al*.^8^.

On days 29 and 30 of the trial, one pig was randomly selected from each pen and sedated by intramuscular injection of Ketamine:Xylazine (20:0 mg/kg BW) and subsequently euthanized by intracardiac injection of 110 mg/kg BW sodium pentobarbital. After euthanizing, pigs were immediately eviscerated from sternum to pubis for collection of intestinal digesta contents, faecal and tissue sub-samples. The whole digestive tract was carefully removed and segmented by clapping to partition the following anatomical parts; jejunum, duodenum, ileum and caecum. Ileal digesta was collected 20 cm from the ileal-caecal junction. Digesta from each segment were collected separately, divided into three sub-samples, transferred into sterile tubes and immediately snap-frozen in liquid nitrogen and stored at −80 °C until further analyses. One sub-sample was freeze-dried and used for bile acids (BA) and neutral sterols (NS) assays. The other two sub-samples were analysed for volatile fatty acids (VFA) concentrations, and DNA extraction and subsequent microbial analyses. The tissue samples were aseptically collected from the jejunum, duodenum, ileum and cecum, flushed with sterile ice cold phosphate buffered saline solution to remove luminal contents and divided into two sub-samples. The first sub-sampled tissue from the ileum and cecum was immediately transferred into sterile tubes, snap-frozen in liquid nitrogen and transferred to −80 °C until used for bacterial genomic DNA extraction and microbial community composition analyses. Tissues from the jejunum, and duodenum, as well as the second tissue sub-samples from the ileum were placed in individual plastic vials and stored in formalin until assessed for histomorphometric characteristics.

### Analysis of the bile acids, neutral sterols and volatile fatty acids

Caecal and ileal digesta samples were thawed on ice and subjected to an acid-base treatment followed by ether extraction and derivatization according to procedures described by Erwin *et al*.^48^. Thereafter, the digesta samples were analysed for VFA concentration using gas chromatography–mass spectrometry (Varian Chromatograph System, model Star 3400; Varian Medical Systems, Palo Alto, CA, USA) using a capillary column (30 m × 0.5 mm; Restek Corp., Belefonte, PA, USA). Freeze-dried faecal, ileal, and caecal samples were ground through a 2 mm screen in a Wiley Mill. Faecal samples were analysed for crude fat according to method described by Ndou *et al*.^8^. The BA and NS were extracted from the digesta and faecal samples according to procedures described by Batta *et al*.^49^.

### Genomic DNA extraction and quality check

Frozen ileal and caecal digesta, and tissue samples were thawed at room temperature. Approximately 200 mg of digesta contents and ~200 mg of mucosa were separately obtained by scrapping the inner wall of each of the ileal and caecal tissue, and used for genomic DNA extraction using ZR Faecal and Tissue DNA extraction kits, respectively (ZYMO Research Corp., Orange, CA, USA). The kit included a bead-beating step for the mechanical lysis of the microbial cells. The DNA concentration was subsequently quantified using a NanoDrop 2000 spectrophotometer (Thermo Scientific, Waltham, MA, USA). Thereafter, DNA samples were adjusted to 20 ng/µl, and quality checked by PCR amplification of the 16 S rRNA gene using universal primers 27 F (5′-GAAGAGTTTGATCATGGCTCAG-3′) and 342 R (5′-CTGCTGCCTCCCGTAG-3′) as described by Khafipour *et al*.^50^. The amplicons were verified by agarose gel electrophoresis.

### Library construction and Illumina sequencing

Library construction and Illumina sequencing were performed as described by Derakhshani *et al*.^51^. In brief, the V4 region of 16 S rRNA was targeted for PCR amplification using modified F515/R806 primers^52^. The reverse PCR primer was indexed with 12-base Golay barcodes allowing for multiplexing of samples. PCR reaction for each sample was performed in duplicate and contained 1.0 µl of pre-normalized DNA, 1.0 µl of each forward and reverse primers (10 µM), 12 µl HPLC grade water (Fisher Scientific, Ottawa, ON, Canada) and 10 µl 5 Prime Hot MasterMix (5 Prime, Inc., Gaithersburg, MD, USA). Reactions consisted of an initial denaturing step at 94 °C for 3 min followed by 35 amplification cycles at 94 °C for 45 sec, 50 °C for 60 sec, and 72 °C for 90 sec; finalized by an extension step at 72 °C for 10 min in an Eppendorf Mastercycler pro (Eppendorf, Hamburg, Germany). PCR products were then purified using ZR-96 DNA Clean-up Kit (ZYMO Research, Irvine, CA, USA) to remove primers, dNTPs and reaction components. The V4 library was then generated by pooling 200 ng of each sample, quantified by Picogreen dsDNA (Invitrogen, Burlington, On, Canada). This was followed by multiple dilution steps using pre-chilled hybridization buffer (HT1) (Illumina, San Diego, CA, USA) to bring the pooled amplicons to a final concentration of 5 pM, measured by Qubit 2.0 Fluorometer (Life technologies, Burlington, ON, Canada). Finally, 15% of PhiX control library was spiked into the amplicon pool to improve the unbalanced and biased base composition, a known characteristic of low diversity 16 S rRNA libraries. Customized sequencing primers for read1 (5′-TATGGTAATTGTGTGCCAGCMGCCGCGGTAA-3′), read2 (5′-AGTCAGTCAGCCGGACTACHVGGGTWTCTAAT-3′) and index read (5′-ATTAGAWACCCBDGTAGTCCGGCTGACTGACT-3′) were synthesized and purified by polyacrylamide gel electrophoresis (Integrated DNA Technologies, Coralville, IA, USA) and added to the MiSeq Reagent Kit V2 (300-cycle) (Illumina, San Diego, CA, USA). The 150 paired-end sequencing reaction was performed on a MiSeq platform (Illumina, San Diego, CA, USA) at the Gut Microbiome and Large Animal Biosecurity Laboratories, Department of Animal Science, University of Manitoba, Canada. The sequencing data were subjected to the Sequence Read Achive (SRA) of NCBA (http://www.ncbi.nlm.nih.gov/sra) and can be assessed using access number SRR5226958. Hierarchical clustering analysis was performed using R (3.3.1 version)^53^ to show a visual interpretation heat map of the similarity of bacterial taxa based on treatment. Normalized relative abundance of bacterial taxa (row normalize length transformation, PAST, version 2.17) was used to for generating the clustering heat map.

### Bioinformatic analyses

Bioinformatic analyses were performed as described by Derakhshani *et al*.^51^. In brief, the PANDAseq assembler was used to merge overlapping paired-end Illumina fastq files^54^. All the sequences with low quality base calling scores as well as those containing uncalled bases (N) in the overlapping region were discarded. The output fastq file was then analysed by downstream computational pipelines of the open source software package QIIME^55^. Assembled reads were demultiplexed according to the barcode sequences, chimeric reads were filtered using UCHIME^56^ and sequences were assigned to Operational Taxonomic Units (OTU) using the QIIME implementation of UCLUST^57^ at 97% pairwise identity threshold. Taxonomies were assigned to the representative sequence of each OTU using RDP classifier^58^ and aligned with the Greengenes Core reference database^59^ using PyNAST algorithms^60^. Phylogenetic tree was built with FastTree 2.1.3. Further comparisons between microbial communities were performed according to Price *et al*.^61^.

Within community diversity (α-diversity) was computed using QIIME. Alpha rarefaction curve was generated using Chao 1 estimator of species richness^62^ with ten sampling repetitions at each sampling depth. An even depth of approximately 15,700 sequences per sample was used for calculation of richness and diversity indices. For comparison of microbial community composition between samples, the β-diversity was measured by calculating the weighted and unweighted UniFrac and Bray-Curtis distances using QIIME default scripts^63^. β-diversity among treatments were visualized using non-metric multidimensional scaling (nMDS) ordination plots that were generated using R software (3.1.0) by employing Bray-Curtis similarity matrices with a conventional cut-off of <0.2 for the stress value. Each data point on the graph represents one sample. The spatial distance between points in the plot was interpreted as the relative difference in the bacterial community composition; thus, points that were closer were more similar than points that were more distant. To assess the statistical differences in β-diversity of bacterial communities among treatment groups, permutational multivariate analysis of variance (PERMANOVA) was performed to calculate *P*-values^64^.

Finally, the open source software PICRUSt (phylogenetic investigation of communities by reconstruction of unobserved states)^65^ was used to predict functional genes of the classified members of the ileal and caecal digesta and mucosa-associated microbiota (resulting from reference based OTU picking against Greengenes database). Predicted genes were then hierarchically clustered and categorized using the Kyoto Encyclopedia of Genes and Genomes (KEGG)^66^ orthologs (KOs) and pathways (levels 1–3).

### Calculations and statistical analyses

To account for differences in mean pig weight between treatments^67^, the average daily feed intake and average weight gain were scaled to live-weight to give SFI and SADG, respectively^68,69^. The SFI was calculated as g of feed per kg BW per day. The SADG was calculated as g of weight gained per kg body weight per day. The coefficient of apparent total tract digestibility (CATTD) of fat was calculated relative to TiO_2_ concentration, as described by Ndou *et al*.^70^.

All other statistical analyses were performed using SAS version 9.4 (SAS, Institute, Inc., Cary, NC, 2009). The UNIVARIATE procedure was used to test for outliers and homogeneity of variances among treatments. Normally distributed data were analysed using a MIXED model. Non-normally distributed data were analysed using GLIMMIX procedure using the negative binomial or Poison distributions. The goodness of fit for each distribution was determined using Pearson chi-square/DF ratio with values closer to 1 considered better. The models accounted for the effects of diet on growth performance, intake of dietary components, histomorphometric characteristics, ATTD of fat, blood lipids, VFA, BA, NS and α-diversity indices of bacterial communities and communities composition. Pen was considered as the experimental unit. Comparisons of means were performed using the Tukey-Kramer honest significant difference test. Significant differences among means were declared at *P* < 0.05, and trends declared for *P* values between 0.05 and 0.10. All the phyla were divided into two groups of abundant, above 1% of the community, and low-abundance, below 1% of the community.

Statistical analyses on the proportion of functional genes and pathways was performed using Linear discriminant analysis (LDA) effect size (LEfSe)^71^, a software principally developed to discover metagenomics biomarkers. Analyses included the non-parametric factorial Kruskal-Wallis (KW) sum rank test^72^, followed by LDA to estimate the effect size of each differentially abundant feature. The threshold on the logarithmic LDA score for discriminative features was set at 2.0, so that features with at least 100-fold shift were considered significant.

Associations between bacterial taxa with an abundance above ≥0.05% of the community and SCFA (acetate, propionate, butyrate), BA (cholic acid; chenodeoxycholic acid; deoxycholic acid; isodeoxycholic acid; lithocholic acid and ursodeoxycholic acid), blood lipids (total cholesterol and triglycerides) and fat digestibility were assessed using non-parametric Spearman’s rank correlation (JMP, Version 10; SAS Institute Inc., Cary, NC, USA). For each correlation, correlation coefficient (Spearman’s Rho) and *P* value were obtained. The correlation coefficient values ranged from −1 to +1 with the upper limit values indicating the strength of the relationship, while positive and negative symbols indicating the direction of association.

## Electronic supplementary material

Supplementary information

## References

[1] Tan J (2014). The role of short-chain fatty acids in health and disease. Adv. Immunol..

[2] Jha R, Berrocoso JFD (2016). Dietary fibre and protein fermentation in the intestine of swine and their interactive effects on gut health and on the environment: A review. Anim. Feed Sci. Tech..

[3] Ferrebee, C. F. & Dawson, P. A. Metabolic effects of intestinal absorption and enterohepatic cycling of bile acids. *Acta Pharmaceutica SinicaB*, https://doi.org/10.1016 (2015). 10.1016/j.apsb.2015.01.001 PMC4629214 26579438

[4] Nie Y, Hu J, Yan X (2015). Cross-talk between bile acids and intestinal microbiota in host metabolism and health. J. Zhejiang Univ-Sci. B. (Biomed. and Biotechnol.).

[5] Wahlstroom A, Sayin SI, Marschall HU, Backhed F (2016). Intestinal crosstalk between bile acids and microbiota and its impact on host metabolism. Cell Metab..

[6] Bakker, G. C. M. Interaction between carbohydrates and fat in pigs. PhD Diss. Wageningen Agricultural Univ., The Netherlands (1996).

[7] Gutierrez NA, Kerr BJ, Patience JF (2013). Effect of insoluble-low fermentable fibre from corn-ethanol distillation origin on energy, fibre, and amino acid digestibility, hindgut degradability of fibre, and growth performance of pigs. J. Anim. Sci..

[8] Ndou SP, Kiarie E, Thandapilly SJ, Ames N, Nyachoti CM (2017). Flaxseed meal and oat hulls supplementation modulates growth performance, blood lipids, intestinal fermentation, bile acids, and neutral sterols in growing pigs fed corn-soybean meal-based diets. J. Anim. Sci..

[9] Pauletti PM, Arau´ jo AR, Young MCM, Giesbrecht AM, Bolzani VS (2000). Nor-Lignans from the leaves of Styrax ferrugineus (Styracaceae) with antibacterial and antifungal activity. Phytochemistry.

[10] Kankaanpaa PE, Salminen SJ, Isolauri E, Lee YK (2001). The influence of polyunsaturated fatty acids on probiotic growth and adhesion. FEMS Microbiol. Lett..

[11] Kim JC, Mullan BP, Hampson DJ, Pluske JR (2008). Addition of oat hulls to an extruded rice-based diet for weaner pigs ameliorates the incidence of diarrhoea and reduces indices of protein fermentation in the gastrointestinal tract. Br. J. Nutr..

[12] Jimenez-Moreno E, Frikha M, de Coca-Sinova A, Garcia J, Mateos GG (2009). Oat hulls and sugar beet pulp in diets for broilers 1. Effects on growth performance and nutrient digestibility. Anim Feed Sci Technol.

[13] Kim JW, Ndou SP, Mejicanos GA, Nyachoti CM (2017). Standardized total tract digestibility of phosphorus in flaxseed meal fed to growing and finishing pigs without or with phytase supplementation1. J. Anim. Sci..

[14] Kiarie E, Nyachoti CM, Slominski BA, Blank G (2007). Growth performance, gastrointestinal microbial activity, and nutrient digestibility in early-weaned pigs fed diets containing flaxseed and carbohydrase enzyme. J. Anim. Sci..

[15] Lopez E, Latorre MA, Valencia DG, Lazaro R, Mateos GG (2003). Inclusion of oat hulls in diets for piglets based on native or cooked cereals. J. Anim. Sci..

[16] Mateos GG, Lo´pez E, Latorre MA, Vicente B, Lazaro RP (2007). The effect of inclusion of oat hulls in piglet diets based on raw or cooked rice and maize. Anim. Feed Sci. Technol..

[17] Lagkouvardos I (2015). Gut metabolites and bacterial community networks during a pilot intervention study with flaxseeds in healthy adult men. Mol. Nutr. Food Res..

[18] Heo JM (2013). Gastrointestinal health and function in weaned pigs: a review of feeding strategies to control post-weaning diarrhoea without using in-feed antimicrobial compounds. J. Anim. Physiol. Anim. Nutr..

[19] Jayaraman, B., Htoo, J. K. & Nyachoti, C. M. Effects of different dietary tryptophan: lysine ratio and sanitary conditions on growth performance, plasma urea nitrogen, serum haptoglobin and ileal histomorphology of weaned pigs. *Anim*. *Sci*. *J*. 763–771 (2016). 10.1111/asj.12695 27677533

[20] Chen XY, Woodward A, Zijlstra RT, Gänzle MG (2014). 2014. Exopolysaccharides synthesized by Lactobacillus reuteri protect against enterotoxigenic Escherichia coli in piglets. Appl. Environ. Microbiol..

[21] Le MHA (2016). Effects of feeding fermented wheat with lactobacillus reuteri on gut morphology, intestinal fermentation, nutrient digestibility, and growth performance in weaned pigs 1. J. Anim. Sci..

[22] Nyachoti CM, Omogbenigun FO, Rademacher M, Blank G (2006). Performance responses and indicators of gastrointestinal health in early-weaned pigs fed low protein amino acid-supplemented diets. J. Anim. Sci..

[23] Vente-Spreeuwenberg MAM, Verdonk JMAJ, Beynen AC, Verstegen MWA (2003). Interrelationship between gut morphology and faeces consistency in newly weaned piglets. Anim. Sci..

[24] Otles S, Ozgoz S (2014). Health effects of dietary fibre. Acta Sci. Pol. Technol. Aliment..

[25] Stanogias G, Pearce GR (1985). The digestion of fibre by pig. 2. Volatile fatty acid concentration in large intestine digesta. Br. J. Nutr..

[26] Hugenholtz F, Mullaney JA, Kleerebezem M, Smidt H, Rosendale DI (2013). Modulation of the microbial fermentation in the gut by fermentable carbohydrates. Bioact. Carbohydr. Dietary Fibre.

[27] Turnbaugh PJ (2009). The effect of diet on the human gut microbiome: a metagenomics analysis in humanized gnotobiotic mice. Sci. Trans. Med..

[28] Devkota S (2012). Dietary-fat-induced taurocholic acid promotes pathobiont expansion and colitis in il10-/- mice. Nature.

[29] Midtvedt T (1974). Microbial bile acid transformation. Am. J. Clin. Nutr..

[30] Hayakawa S (1982). Microbial transformation of bile acids. A unified scheme for bile acid degradation, and hydroxylation of bile acids. Z. Allg. Mikrobiol..

[31] Doerner KC, Takamine F, Lavoie CP, Mallonee DH, Hylemon PB (1997). Assessment of faecal bacteria with bile acid 7α-dehydroxylating activity for the presence of bai-like genes. Appl. Environ. Microbiol..

[32] Begley M, Gahan CG, Hill C (2005). The interaction between bacteria and bile. FEMS Microbiol. Rev..

[33] Ridlon JM, Kang DJ, Hylemon PB (2006). Bile salt biotransformations by human intestinal bacteria. J. Lipid Res..

[34] Ridlon JM, Kang DJ, Hylemon PB, Bajaj JS (2014). Bile acids and the gut microbiome. Curr. Opin. Gastroenterol..

[35] Salvioli G (1982). Bile acid transformation by the intestinal flora and cholesterol saturation in bile. Effects of Streptococcus faecium administration. Digestion.

[36] Mathlouthi N, Lalles JP, Lepercq P, Juste C, Larbier M (2002). Xylanase and β-glucanase supplementation improve conjugated bile acid fraction in intestinal contents and increase villus size of small intestine wall in broiler chickens fed a rye-based diet. J. Anim. Sci..

[37] Archer RH, Chong R, Maddox IS (1982). Hydrolysis of bile acid conjugates by clostridium bifermentans. Eur. J. Appl. Microbiol..

[38] Gilliland SE, Speck ML (1977). Deconjugation of bile acids by intestinal *lactobacilli*. Appl. Environ. Microbiol..

[39] Jones BV, Begley M, Hill C, Gahan CG, Marchesi JR (2008). Functional and comparative metagenomic analysis of bile salt hydrolase activity in the human gut microbiome. Proc. Natl. Acad. Sci. USA.

[40] Soder KJ, Brito AF, Rubano MD, Dell CJ (2012). Effect of incremental flaxseed supplementation of an herbage diet on methane output and ruminal fermentation in continuous culture. J. Dairy Sci..

[41] Berggren AM, Bjorck IME, Nyman MGL (1993). Short-chain fatty acid content and pH in caecum of rats given various sources of carbohydrates. J Sci Food Agric.

[42] Dierick N, Decuypere J (1996). Mode of action of exogenous enzymes in growing pig nutrition. Pig News Info..

[43] Pluske JR, Pethick DW, Hopwood DE, Hampson DJ (2002). Nutritional influences on some major enteric bacterial diseases of pigs. Nutr. Res. Rev..

[44] Belcheva A, Irrazabal T, Robertson SJ (2001). Gut microbial metabolism drives transformation of MSH2-defiacient colon epithelial cells. Cell.

[45] Simpson H, Campbell BJ (2015). Review article: dietary fibre-microbiota interactions. Aliment. Pharmacol. Ther..

[46] Vital M, Howe AC, Tiedje JM (2014). Revealing the bacterial butyrate synthesis pathways by analyzing (meta) genomic data. MBio.

[47] Canadian Council on Animal Care. Guides to the care and use of experimental animals in research, teaching and testing. Ottawa, ON, Canada (2009).

[48] Erwin, E. S., Marco, G. J. & Emery, E. M. Volatile fatty acids analyses of blood and rumen fluid by gas chromatography. *J. Dairy Sci.* **44**, 1768–1771 **61**, 89956–6 (1961).

[49] Batta AK (1999). Highly simplified method for gas-liquid chromatographic quantitation of bile acids and sterols in human stool. J. Lipid Res..

[50] Khafipour E, Li S, Plaizier JC, Krause DO (2009). Rumen microbiome composition determined using two nutritional models of subacute ruminal acidosis. Appl. Environ. Microbiol..

[51] Derakhshani H, Tun HM, Khafipour E (2016). An extended single-index multiplexed 16S rRNA sequencing for microbial community analysis on MiSeq Illumina platforms. J. basic Microbiol..

[52] Caporaso JG (2012). Ultra-high-throughput microbial community analysis on the Illumina HiSeq and MiSeq platforms. The ISME J..

[53] R Development Core Team, R: A Language and Environment for Statistical Computing. Vienna, Austria: the R Foundation for Statistical Computing (2014).

[54] Masella A, Bartram A, Truszkowski J, Brown D, Neufeld J (2012). PANDAseq: paired-end assembler for illumina sequences. BMC Bioinformatics.

[55] Caporaso JG (2010). QIIME allows analysis of high-throughput community sequencing data. Nature methods..

[56] Edgar RC, Haas BJ, Clemente JC, Quince C, Knight R (2011). UCHIME improves sensitivity and speed of chimera detection. Bioinformatics.

[57] Edgar RC (2010). Search and clustering orders of magnitude faster than BLAST. Bioinformatics.

[58] Wang Q, Garrity GM, Tiedje JM, Cole JR (2007). Naive Bayesian classifier for rapid assignment of rRNA sequences into the new bacterial taxonomy. Appl. Environ. Microbiol..

[59] DeSantis TZ (2006). Greengenes, a chimera-checked 16S rRNA gene database and workbench compatible with ARB. Appl. Environ. Microbiol..

[60] Caporaso JG (2010). PyNAST: a flexible tool for aligning sequences to a template alignment. Bioinformatics.

[61] Price MN, Dehal PS, Arkin AP (2010). FastTree 2–approximately maximum-likelihood trees for large alignments. PloS one.

[62] Chao, A. Nonparametric estimation of the number of classes in a population. *Scand*. *J*. *Stat*. 265-70 (1984).

[63] Lozupone C, Knight R (2005). UniFrac: a new phylogenetic method for comparing microbial communities. Appl. Environ. Microbiol..

[64] Anderson M. *PERMANOVA*: a FORTRAN Computer Program for Permutational Multivariate Analysis of Variance (Department of Statistics, University of Auckland, 2005).

[65] Langille MG (2013). Predictive functional profiling of microbial communities using 16S rRNA marker gene sequences. Nat. Biotechnol..

[66] Kanehisa M, Goto S (2000). Kegg: kyoto encyclopedia of genes and genomes. Nucleic Acids Res..

[67] Ndou S, Gous R, Chimonyo M (2013). Prediction of scaled feed intake in weaner pigs using physico-chemical properties of fibrous feeds. Br. J. Nutr..

[68] Kyriazakis I, Emmans G (1995). The voluntary feed intake of pigs given feeds based on wheat bran, dried citrus pulp and grass meal, in relation to measurements of feed bulk. Br. J. Nutr..

[69] Ndou SP, Bakare AG, Chimonyo M (2013). Prediction of voluntary feed intake from physicochemical properties of bulky feeds in finishing pigs. Livest. Sci..

[70] Ndou SP (2015). Comparative efficacy of xylanases on growth performance and digestibility in growing pigs fed wheat and wheat bran-or corn and corn DDGS-based diets supplemented with phytase. Anim. Feed Sci. Technol..

[71] Segata N (2011). Metagenomic biomarker discovery and explanation. Genome Biol..

[72] Kruskal WH, Wallis WA (1952). Use of ranks in one-criterion variance analysis. J. Am. Stat. Assoc..

