# Supplementary material for: Dietary supplementation with flaxseed meal and oat hulls modulates intestinal histomorphometric characteristics, digesta- and mucosa-associated microbiota in pigs
Source: Sci Rep. 2018 Apr 12;8:5880. doi: 10.1038/s41598-018-24043-5 (PMC5897541; doi:10.1038/s41598-018-24043-5)
Supplement: Supplementary file 1 — Supplementary information [file 41598_2018_24043_MOESM1_ESM.pdf]

Supplementary information

Running tittle: Dietary fiber alters intestinal milieu

Dietary supplementation with flaxseed meal and oat hulls modulates intestinal histomorphometric characteristics, digesta- and mucosa-associated microbiota in pigs

S.P. Ndou[a], H.M. Tun[a], E. Kiarie[a,b], M.C. Walsh[d], E. Khafipour[a,c,*] and C.M. Nyachoti[a,*]

[a]*Department of Animal Science, University of Manitoba, Winnipeg, MB, Canada. R3T 2N2*

[b]*Department of Animal Biosciences, University of Guelph, Guelph, ON, Canada, N1G 2W1*

[c]*Department of Medical Microbiology, University of Manitoba, Winnipeg, MB, Canada. R3T 2N2*

[d]*DuPont Industrial Biosciences-Danisco Animal Nutrition, Marlborough, Wiltshire, United Kingdom SN8 1XN*

*Co-corresponding author:

Dr. Martin Nyachoti, Tel: +1-204-474-7323; Fax: +1-204-474-7628; EMail: Martin_Nyachoti@umanitoba.ca

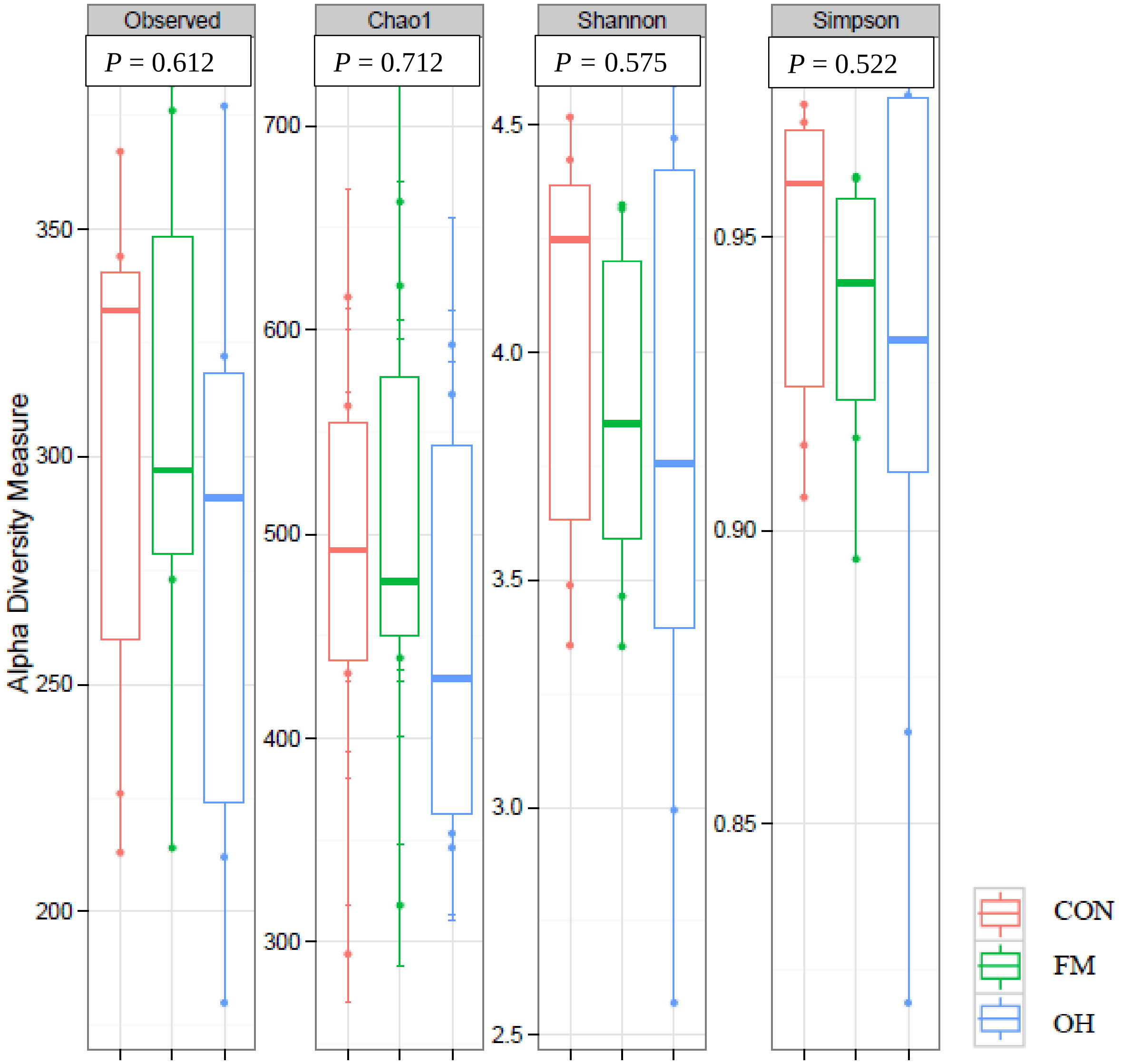

Supplementary Figure S1: Alpha-diversity indices of microbiota in ileal digesta of pigs fed with control (CON), flaxseed meal (FM) and oat hulls (OH) diets

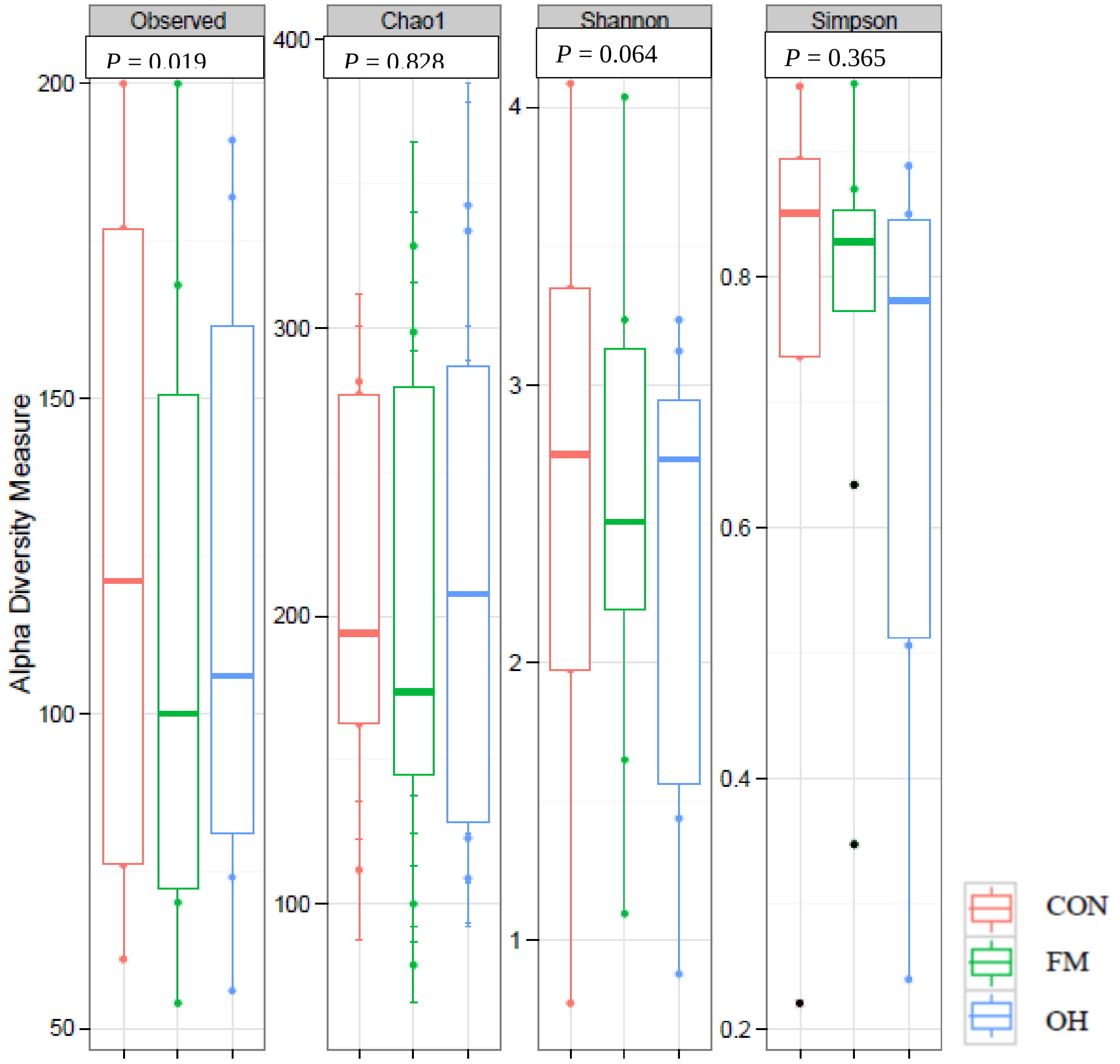

Supplementary Figure S2: Alpha-diversity indices of ileal mucosa-associated microbiota of pigs fed with control (CON), flaxseed meal (FM) and oat hulls (OH) diets

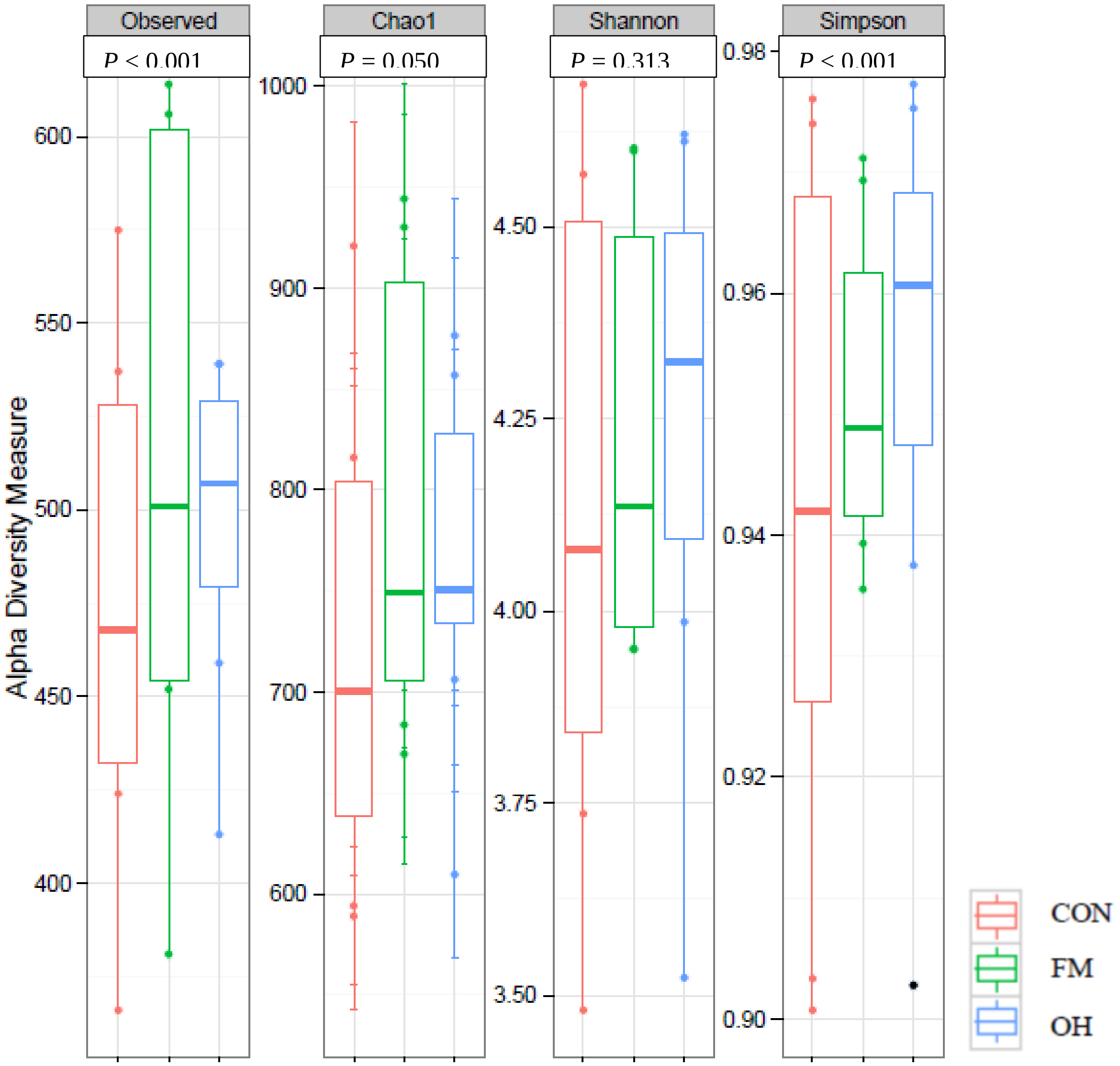

Supplementary Figure S3: Alpha-diversity indices of microbiota in cecal digesta of pigs fed with control (CON), flaxseed meal (FM) and oat hulls (OH) diets

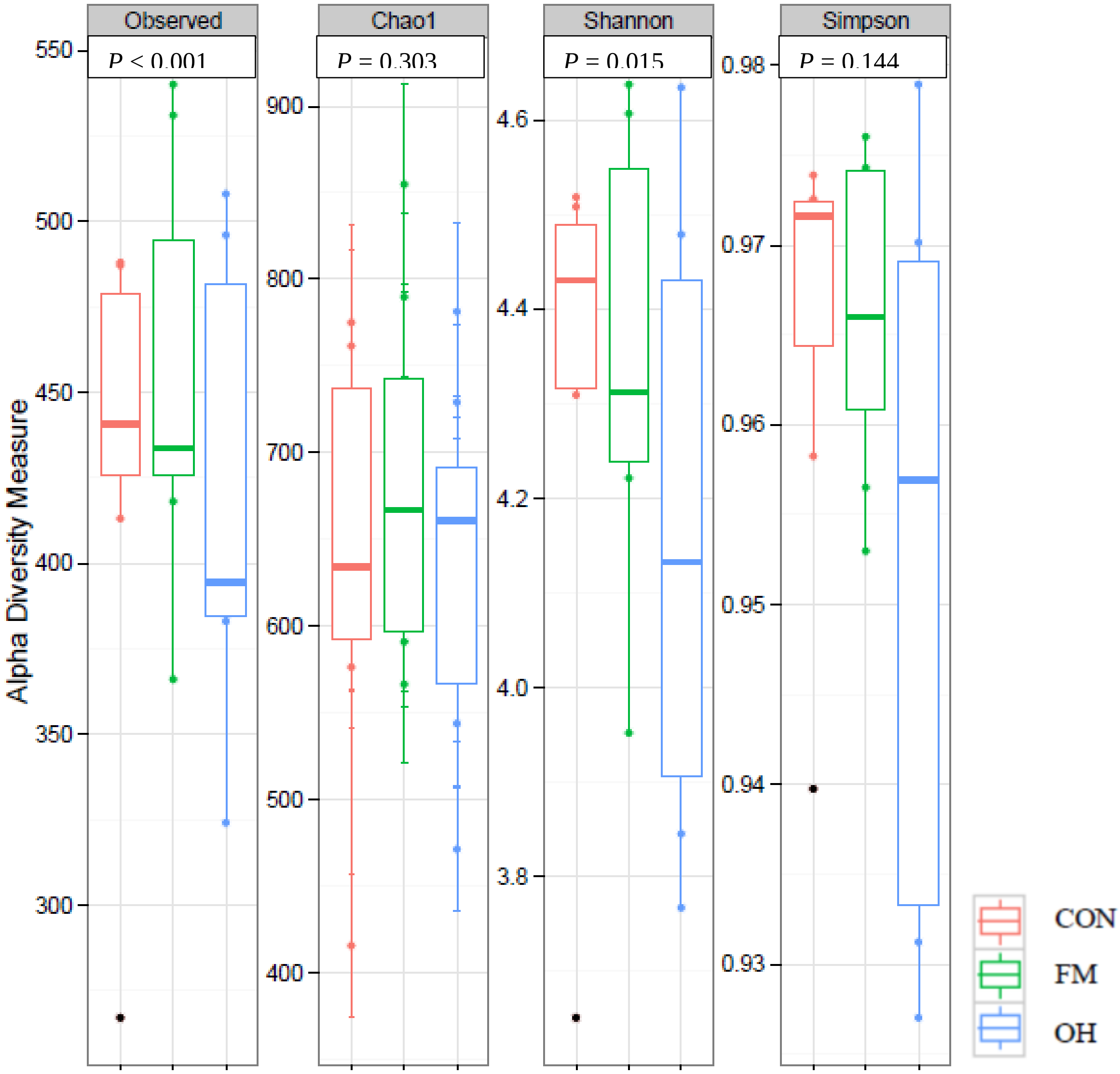

Supplementary Figure S4: Alpha-diversity indices of cecal mucosa-associated microbiota of pigs fed with control (CON), flaxseed meal (FM) and oat hulls (OH) diets.

Table S1. Ingredient composition and calculated provisions of the control, flaxseed meal and oat hulls diets[1]

	Diet[1]		
	CON	FM	OH
Ingredients			
Corn	64.53	57.40	52.99
Oat hulls	-	-	10.00
Flaxseed meal	-	12.00	-
Soybean meal, 44 % CP	31.00	25.49	31.500
Vegetable oil	1.354	2.044	2.390
Limestone	0.683	0.667	0.640
Monocalcium phosphate	0.752	0.637	0.750
Salt	0.35	0.35	0.35
Vitamin-mineral premix[2]	1.00	1.00	1.00
L-Lysine HCl	-	0.086	0.010
DL-Methionine	0.031	0.010	0.060
Threonine	-	0.012	0.010
Titanium dioxide	0.30	0.30	0.30
Calculated dietary provisions[3]			
ME kcal/kg	3,330	3,334	3,326
CP, %	18.10	18.70	18.01
SID Lys, %	0.99	0.99	0.99
SID Met, %	0.30	0.31	0.30
SID Met + Cys, %	0.58	0.61	0.55
SID Thr, %	0.62	0.60	0.60
SID Trp, %	0.21	0.17	0.18

[1]CON = control diet; FM = flaxseed meal-containing diet; OH = oat hulls-containing diet.

[2]Provided the following nutrients per kg of air-dry diet: 8,250 IU retinol (Vitamin A); 200 IU cholecalciferol (Vitamin D_3); 40 UI α-tocopherol (Vitamin E); 4 mg Vitamin K; 1.5 mg Vitamin B_1; 7 mg Vitamin B_2; 2.5 mg Vitamin B_6; 25 µg Vitamin B_{12}; 14 mg calcium pantothenate; 2 mg folic acid; 21 mg niacin (Vitamin B_3); and 200 µg biotin (Vitamin B_7). Minerals: 15 mg Cu (as copper sulphate); 0.4 mg iodine (as potassium iodine); 120 mg iron (as ferrous sulphate); 20 mg Mn (as manganese oxide); 0.3 mg Se (as sodium selenite); 110 mg Zn (as zinc oxide).

[3]The nutrient compositions were calculated based on ingredient composition values from NRC (2012) and our laboratory.

Table S2. Analyzed composition of the experimental diets

Item	Diets[1]		
	CON	FM	OH
Chemical composition			
Crude protein (N × 6.25), %	19.14	19.33	19.24
Gross energy, kcal/kg	3879	3886	3977
Fat content, %	4.58	5.32	5.94
Calcium	0.61	0.62	0.60
Phosphorus	0.58	0.63	0.58
Neutral detergent fiber, %	9.37	17.9	18.6
Acid detergent fiber, %	3.6	4.8	7.2
NSP constituent sugars, %[2]			
Total NSP	9.48 (0.80)	13.3 (3.50)	15.0 (1.79)
Arabinose	1.80 (0.16)	2.20 (0.36)	1.84 (0.06)
Xylose	1.83 (0.06)	2.76 (0.79)	4.81 (0.38)
Mannose	0.26 (0.00)	0.23 (0.06)	0.22 (0.01)
Galactose	1.50 (0.15)	1.75 (0.50)	1.30 (0.06)
Glucose	2.78 (0.43)	4.26 (1.29)	5.60 (0.94)
Uronic acids	1.32 (0.12)	2.10 (0.50)	1.20 (0.35)
Physical properties			
Bulk density, g/ml	1.56	1.43	1.36
Swelling capacity, ml/g	2.67	4.51	2.85
Water holding capacity, ml/g	3.31	5.98	4.52
[3]Particle size distribution, %			
>1700 µm	19.8	20.6	18.2
> 850 µm	19.3	16.6	22.7
< 600 µm	61.0	62.8	59.1

[1]CON = control diet; FM = flaxseed meal-containing diet; OH = oat hulls-containing diet. NSP = non-starch polysaccharides.

[2]Values in parentheses are for the soluble proportions of the NSP.

[3]100 g of sample size for each diet.

Table S3. Intake of dietary components and growth performance of growing pigs fed the control, flaxseed meal and oat hulls diets[1]

[3]Item	[2]Diet			SEM[3]	*P-value*
	CON	FM	OH		
Intake of dietary components					
Gross energy, MJ/d	30.02[b]	26.80[c]	32.21[a]	0.724	<0.001
Metabolizable energy, MJ/g	25.86[a]	22.79[b]	24.84[a]	0.602	0.006
Crude protein, g/d	354.6	342.8	354.3	8.582	0.551
Fat, g/d	84.7[b]	87.8[b]	109.4[a]	2.264	<0.001
Soluble NSP[4], g/d	14.80[c]	57.69[a]	32.96[b]	0.939	<0.001
Insoluble NSP, g/d	160.6[b]	161.5[b]	180.5[a]	4.066	0.003
Total NSP, g/d	175.4[c]	219.2[b]	276.2[a]	5.362	<0.001
CATTD of fat[5]	0.924[a]	0.701[c]	0.792[b]	0.038	0.020
Growth performance					
SFI[6], g/kg BW/d	38.46	39.03	39.96	1.02	0.365
SADG[7], g/kg BW/d	17.74[A]	15.11[B]	16.90[A]	0.76	0.064
FCR[8]	2.16	2.58	2.36	0.46	0.106

[abc]Within a row, means with unlike superscripts differ ($P < 0.05$).

[1]Least square mean values for intake and growth performance were calculated from data and dietary composition assays reported by Ndou et al., 2017.

[2]CON, Control diet; FM, Flaxseed meal-containing diet; OH, Oat hulls-containing diet.

[3]Standard error of treatment mean (n = 8 replicates per treatment).

[4]NSP, Non-starch polysaccharides.

[5]CATTD, Coefficient of apparent total tract digestibility of fat.

[6]SFI, Scaled feed intake.

[7]SADG, Scaled average daily weight gain.

[8]FCR, Feed conversion ratio.

Table S4. Histomorphometric characteristics of intestinal tissue in growing pigs fed experimental diets[1]

[3]Item	[2]Diet			[4]SEM	*P*-value[5]
	CON	FM	OH		
Duodenum					
VH, µm	507.7^{a}	471.6^{b}	464.4^{b}	10.29	0.0004^{G}
CD, µm	344.9^{AB}	350.8^{A}	324.6^{B}	8.27	0.0568^{G}
VH:CD	1.47^{A}	1.35^{B}	1.47^{A}	0.038	0.0732^{G}
Jejunum					
VH, µm	450.1^{b}	523.1^{a}	458.5^{b}	6.77	$<0.0001^{P}$
CD, µm	307.4	319.1	301.9	3.50	0.7892^{G}
VH:CD	1.46^{b}	1.64^{a}	1.52^{b}	0.027	$<0.0001^{NB}$
Ileum					
VH, µm	471.4^{a}	420.8^{b}	404.3^{c}	4.78	$<0.001^{P}$
CD, µm	286.2	295.5	278.1	12.64	0.3683^{NB}
VH:CD	1.69^{a}	1.43^{b}	1.46^{ab}	0.106	0.0369^{G}

[1]Least square mean values for histomorphometric characteristics.

[abc]Within a row, means with unlike superscripts differ ($P < 0.05$)

[ABC]Within a row, means with unlike superscripts differ ($P \leq 0.10$).

[2]CON, Control diet; FM, Flaxseed meal-containing diet; OH, Oat hulls-containing diet.

[3]VH, villi height; CD, crypt depth; VCR, villi height to crypt depth ratio.

[4]Standard error of treatment mean (n = 8 replicates per treatment).

[5]Methods of analysis denoted by ^{G} (Gaussian), ^{NB} (Negative Binomial) and ^{P} (Poisson).

Table S5. Effect of diet on alpha-diversity indices[1] of cecal and ileal digesta microbiota of pigs fed control diet (CON), flaxseed meal (FM) and oat hulls (OH)

Item	Ileum					Cecum				
	[2]Diet					[2]Diet				
	CON	FM	OH	SEM[3]	*P*-value	CON	FM	OH	SEM[3]	*P*-value
Observed_species[4]	303	304	277	60.3	0.612	462^{b}	595^{a}	510^{b}	23.85	<0.001
Coverage[5]	0.955	0.955	0.959	0.010	0.673	0.975	0.973	0.974	0.0016	0.799
Chao1[6]	485	494	453	104.7	0.712	722^{C}	805^{A}	769^{B}	65.3	0.050
Shannon[7]	5.827	5.583	5.418	0.598	0.575	5.700	6.035	6.126	0.328	0.313
Simpson[8]	0.947	0.937	0.925	0.021	0.522	0.942^{b}	0.951^{a}	0.954^{a}	0.0003	<0.001

[1]Least square mean values for alpha biodiversity indices.

[abc]Within a row, means with unlike superscripts differ ($P < 0.05$)

[ABC]Within a row, means with unlike superscripts differ ($P \leq 0.10$).

[2]CON, Control diet; FM, Flaxseed meal-containing diet; OH, Oat hulls-containing diet.

[3]Standard error of treatment mean (n = 8 replicates per treatment).

[4]Observed_species is the unique OTUs (97% distance) found in the samples.

[5]Coverage estimates the percentage of the total species sequenced in samples.

[6]Chao richness index estimates the number of species or OTU presenting in samples.

[7,8] Indices of microbiota diversity, which take into account species richness and abundance.

Table S6. Effect of diet on alpha-diversity indices[1] of cecal and ileal mucosa-associated microbiota of pigs fed control diet (CON), flaxseed meal (FM) and oat hulls (OH)

Item	Ileum					Cecum				
	[2]Diet			SEM[3]	*P*-value	[2]Diet			SEM[3]	*P*-value
	CON	FM	OH			CON	FM	OH		
Observed_species[4]	91.6^{b}	151.3^{a}	115.8^{ab}	1487	0.019	393^{b}	504^{a}	445^{ab}	3044	0.002
Coverage[5]	0.973	0.963	0.962	0.021	0.246	0.978	0.967	0.978	0.0016	0.671
Chao1[6]	216.1	223.4	201.6	8.288	0.828	553^{b}	683^{a}	638^{ab}	9.103	0.039
Shannon[7]	2.323^{B}	3.945^{A}	3.611^{B}	0.601	0.064	6.249^{a}	6.279^{a}	5.761^{b}	0.130	0.015
Simpson[8]	0.770	0.840	0.732	0.022	0.365	0.966	0.966	0.953	0.0021	0.144

[1]Least square mean values for alpha biodiversity indices.

[abc]Within a row, means with unlike superscripts differ ($P < 0.05$)

[ABC]Within a row, means with unlike superscripts differ ($P \leq 0.10$).

[2]CON, Control diet; FM, Flaxseed meal-containing diet; OH, Oat hulls-containing diet.

[3]Standard error of treatment mean (n = 8 replicates per treatment).

[4]Observed_species is the unique OTUs (97% distance) found in the samples.

[5]Coverage estimates the percentage of the total species sequenced in samples.

[6]Chao richness index estimates the number of species or OTU presenting in samples.

[7,8] Indices of microbiota diversity, which take into account species richness and abundance.

Table S7. Relative abundance of bacterial phyla in ileal digesta-associated microbiota of pigs fed control diet (CON), flaxseed meal (FM) and oat hulls (OH) †

Phyla present	[1]Diets			SEM[2]	*P-value*[3]
	CON	FM	OH		
------------------------------------Abundant Phylum (>1% of population)-------------------------					
Firmicutes	60.21 ± 5.32[B]	73.23 ± 3.84[A]	72.92 ± 2.85[A]	6.612	0.081[G]
Bacteroidetes	25.72 ± 4.22	20.05 ± 8.59	22.98 ± 1.12	5.321	0.772[P]
Proteobacteria	1.12 ± 0.24[b]	5.01 ± 0.33[a]	2.82 ± 0.68[a]	0.097	<0.01[G]
Unclassified	11.25 ± 4.23[a]	1.05 ± 4.23[b]	2.85 ± 3.53[b]	2.361	0.023[G]
----------------------------------Low-abundant Phylum (<1% of population)---------------------					
Spirochaetes	0.42 ± 0.22	0.35 ± 1.29	0.58 ± 0.42	0.831	0.362[P]
Actinobacteria	0.21 ± 0.05	0.12 ± 0.08	0.33 ± 0.05	0.003	0.51[G]
Cyanobacteria	0.02 ± 0.20	0.02 ± 0.02	0.03± 0.03	0.011	0.67[G]
Tenericutes	0.01 ± 0.24	0.01 ± 0.33	0.02 ± 0.18	0.017	0.61[G]
Elusimicrobia	0.01 ± 0.05	0.02 ± 0.18	0.01 ± 0.25	0.003	0.78[P]
Fibrobacteres	0.01 ± 0.20	0.02 ± 0.12	0.03± 0.43	0.011	0.67[G]
Synergistetes	0.01 ± 0.05	0.02 ± 0.08	0.01 ± 0.05	0.003	0.88[G]

[1]CON, Control diet; FM, Flaxseed meal-containing diet; OH, Oat hulls-containing diet;
[2]SEM, standard error of means;
[†]Least square mean values (n = 8 replicates per treatment).
[abc]Mean values within a row with unlike superscripts are significantly different ($P < 0.05$).
[ABC]Mean values within a row with unlike superscripts are significantly different ($0.05 < P < 0.10$).
[3]Methods of analysis denoted by [G](Gaussian), and [P](Poison).

Table S8. Relative abundance of bacterial phyla in ileal mucosa-associated microbiota of pigs fed control diet (CON), flaxseed meal (FM) and oat hulls (OH) †

Phyla present	[1]Diets			[2]SEM	*P-value*[3]
	CON	FM	OH		
--Abundant Phylum (>1% of population)--------------------------					
Bacteroidetes	13.22 ± 1.54^{ab}	17.26 ± 2.56^{a}	9.01 ± 2.88^{b}	3.251	<0.001^{G}
Firmicutes	80.04 ± 5.97^{a}	66.25 ± 4.74^{b}	65.01 ± 4.81^{b}	6.872	0.001^{P}
Proteobacteria	5.08 ± 6.61^{b}	14.09 ± 2.36^{ab}	17.05 ± 3.13^{a}	3.924	0.100^{NB}
Unclassified	1.16 ±1.69	2.11 ± 3.65	8.53 ± 6.59	6.542	0.361^{G}
---------------------------------------Low abundant Phylum (<1% of population)----------------------					
Actinobacteria	0.41 ± 0.62^{A}	0.03 ± 0.09^{C}	0.11 ± 0.25^{B}	0.002	0.050^{P}
Spirochaetes	0.11 ± 0.15	0.05 ± 0.08	0.07 ± 0.11	0.051	0.551^{G}
Cyanobacteria	0.09 ± 0.07	0.08 ± 0.08	0.10 ± 0.11	0.055	0.961^{G}
Deferribacteres	0.01 ± 0.02^{b}	0.02 ± 0.03^{b}	0.06 ± 0.03^{a}	0.015	0.011^{G}
Synergistetes	0.02 ± 0.03	0.01 ± 0.02	0.01 ± 0.02	0.013	0.771^{G}
WPS-2	0.01 ± 0.15	0.02 ± 0.02	0.02 ± 0.11	0.031	0.365^{G}
Elusimicrobia	0.01 ± 0.11	0.02 ± 0.08	0.03 ± 0.11	0.042	0.111^{G}

[1]CON, Control diet; FM, Flaxseed meal-containing diet; OH, Oat hulls-containing diet;
[2]SEM, standard error of means.
†Least square mean values (n = 8 replicates per treatment).
^{abc}Mean values within a row with unlike superscripts are significantly different ($P < 0.05$).
^{ABC}Mean values within a row with unlike superscripts are significantly different ($P < 0.10$).
[3]Methods of analysis denoted by ^{G} (Gaussian), ^{NB} (Negative Binomial) and ^{p} (Poison).

Table S9. Phylogenetic composition of bacterial genus from illumina-sequencing of 16S rRNA sequences in ileal digesta of pigs fed experimental diets

Order (O), Family (F), genus (G)	Diets[1]			SEM[2]	*P-value*[3]
	CON	FM	OH		
……………………………………Actinobacteria (P)………………………………………….					
Unclassified Coriobacteriaceae (F)	0.104^{AB}	0.154^{A}	0.079^{B}	0.031	0.068^{G}
……………………………………Bacteroidetes (P)………………………………………….					
Unclassified Bacteroidales (O)	0.543	1.024	0.543	0.434	0.614^{P}
Bacteroides	0.117	0.370	0.100	0.137	0.114^{G}
Prevotella	22.44^{a}	14.47^{b}	16.85^{ab}	3.207	0.004^{P}
Parabacteroides	0.067	0.083	0.046	0.040	0.659^{G}
CF231	1.15	1.36	0.99	0.425	0.799^{P}
YRC22	0.083^{b}	0.342^{a}	0.163^{ab}	0.099	0.048^{G}
……………………………………Firmicutes (P)………………………………………….					
Lactobacillus	17.96^{b}	28.70^{a}	23.36^{ab}	1.597	0.002^{P}
Streptococcus	5.56^{B}	9.08^{A}	7.09^{AB}	1.647	0.087^{NB}
Unclassified Clostridiales (O)	3.12^{b}	4.02^{a}	1.82^{c}	0.225	0.015^{G}
Unclassified Clostridiaceae (F)	3.94^{b}	8.54^{a}	4.40^{b}	1.681	0.002^{P}
Clostridium	0.395	0.355	0.267	0.142	0.658^{G}
Blautia	2.16	1.25	2.58	0.711	0.195^{P}
Coprococcus	0.531^{ab}	0.357^{b}	0.754^{a}	0.142	0.037^{G}
Dorea	0.305^{B}	0.395^{AB}	0.658^{A}	0.146	0.065^{G}
Roseburia	0.273	0.322	0.360	0.102	0.695^{G}
Unclassified Ruminococcaceae (F)	3.65	3.16	3.74	0.568	0.554^{G}
Ruminococcus	0.518	0.478	0.783	0.239	0.698^{P}
Faecalibacterium	0.512	0.475	0.433	0.126	0.823^{G}
Oscillospira	0.353	0.539	0.383	0.226	0.836^{P}
Peptococcus	0.0708	0.1084	0.1208	0.046	0.536^{G}
Unclassified Veillonellaceae (F)	2.48^{a}	1.62^{ab}	0.283^{b}	0.804	0.024^{NB}
Acidaminococcus	0.500	0.217	0.071	0.119	0.108^{P}
Anaerovibrio	0.842	0.938	0.967	0.265	0.880^{G}
Dialister	0.308	0.138	0.175	0.106	0.262^{G}
Mitsuokella	5.18^{ab}	12.50^{a}	0.133^{b}	4.945	0.031^{NB}
Megasphaera	16.75	15.50	17.41	4.527	0.913^{G}
Phascolarctobacterium	9.33^{a}	2.09^{ab}	1.11^{b}	5.212	<0.001^{NB}
Unclassified Erysipelotrichaceae (F)	0.0666^{AB}	0.0418^{B}	0.108^{A}	0.026	0.055^{G}
Bulleidia	1.32	1.92	0.900	0.924	0.557^{P}
……………………………………Proteobacteria………………………………………….					
Succinivibrio	0.154	0.154	0.146	0.058	0.986^{P}
Unclassified Enterobacteriaceae (F)	0.103^{b}	0.218^{b}	2.575^{a}	0.095	0.047^{NB}

[1]CON, Control diet; FM, Flaxseed meal-containing diet; OH, Oat hulls-containing diet;
[2]SEM, standard error of means (n = 8 replicates per treatment).
[abc]Mean values within a row with unlike superscripts are significantly different (P < 0.05).
[ABC]Mean values within a row with unlike superscripts are significantly different (P < 0.10).
[3]Methods of analysis denoted by G (Gaussian), [NB] (Negative Binomial) and [P] (Poison).

Table S10. Phylogenetic composition of bacterial genus from illumina-sequencing of 16S rRNA sequences in ileal mucosa of pigs fed experimental diets

Order (O), Family (F), Genus	Diets[1]			SEM[2]	*P-value*[3]
	CON	FM	OH		
……………………………………Actinobacteria (P)………..……………………………					
Actinobacteria_unclassified	0.5694	0.0468	0.0103	0.332	0.169^{G}
……………………….……..…...…….Bacteroidetes (P)………..………..…………………..					
Bacteroidales _unclassified (O)	4.70^{b}	24.48^{a}	20.42^{a}	6.788	<0.001^{P}
……………………….…….….……Firmicutes (P)………..…..……………………………..					
Lactobacillus	9.95^{b}	58.83^{a}	59.48^{a}	11.223	<0.001^{NB}
Streptococcus	3.71^{AB}	2.91^{B}	5.57^{A}	1.059	0.083^{P}
Unclassified Clostridiales (O)	2.63^{a}	0.488^{b}	1.93^{ab}	1.101	0.048^{NB}
Unclassified Clostridiaceae (F)	31.38^{a}	0.283^{b}	1.02^{b}	7.591	<0.001^{P}
Unclassified Lachnospiraceae (F)	1.55	0.679	0.925	0.472	0.193^{G}
Unclassified Ruminococcaceae (F)	1.86	1.85	1.13	0.851	0.627^{G}
Unclassified Veillonellaceae (F)	7.53^{a}	2.46^{b}	5.09^{ab}	2.587	0.002^{P}
Veillonella	0.137^{b}	2.79^{a}	0.372^{b}	0.384	0.004^{P}
…………..…………………….………..Proteobacteria (P)……………..………………………					
Campylobacter	0.2088	0.2251	0.2327	0.109	0.973^{P}
Helicobacter	2.2358^{a}	2.4393^{a}	0.2583^{b}	0.939	0.024^{P}

[1]CON, Control diet; FM, Flaxseed meal-containing diet; OH, Oat hulls-containing diet;
[2]SEM, standard error of means;
†Least square mean values (n = 8 replicates per treatment).
[abc]Mean values within a row with unlike superscripts are significantly different ($P < 0.05$).
[ABC]Mean values within a row with unlike superscripts are significantly different ($P < 0.10$).
[3]Methods of analysis denoted by G (Gaussian), [NB] (Negative Binomial) and [P] (Poison).

Table S11. Relative abundance of bacterial phyla in cecal digesta-associated microbiota of pigs fed control diet (CON), flaxseed meal (FM) and oat hulls (OH)

Phyla present	Diets[1]			SEM[2]	*P-value*[3]
	CON	FM	OH		
---------------------------------------Abundant Phylum (>1% of population)---------------------------					
Bacteroidetes	34.71±11.72	29.04±7.91	33.63±3.07	4.762	0.421^{P}
Firmicutes	60.14±1.97^{b}	64.08±0.73^{a}	59.70±0.67^{b}	1.031	<0.001^{G}
Proteobacteria	1.11±0.43^{c}	5.65±0.91^{a}	2.05±0.50^{b}	0.633	0.049^{P}
Unclassified	2.46±0.65	0.04±0.15	4.01±5.63	3.658	0.215^{G}
----------------------------------Low abundant Phylum (<1% of population)-------------------------					
Actinobacteria	0.236±0.06^{A}	0.078±0.06^{B}	0.139±0.051^{AB}	0.007	0.060^{G}
Chlamydiae	0.565±0.01	0.263±0.532	0.273±0.004	0.251	0.592^{P}
Cyanobacteria	0.236±0.16^{a}	0.078±0.031^{b}	0.139±0.115^{ab}	0.060	0.048^{G}
Deferribacteres	0.053±0.01	0.005±0.013	0.027±0.06	0.018	0.351^{G}
Elusimicrobia	0.031±0.05	0.025±0.041	0.052±0.032	0.011	0.123^{P}
Fibrobacteres	0.023±0.02	0.022±0.018	0.044±0.032	0.016	0.344^{G}
Synergistetes	0.028±0.02	0.029±0.017	0.022±0.019	0.010	0.736^{G}
TM7	0.011±0.02	0.006±0.007	0.006±0.009	0.006	0.695^{G}
Tenericates	0.028±0.02^{a}	0.005±0.013^{b}	0.021±0.011^{ab}	0.010	0.039^{G}
WPS-2	0.078±0.07	0.027±0.0122	0.028±0.025	0.030	0.244^{G}

[1]CON, Control diet; FM, Flaxseed meal-containing diet; OH, Oat hulls-containing diet;
[2]SEM, standard error of means;
[†]Least square mean values (n = 8 replicates per treatment);
[abc]Mean values within a row with unlike superscripts are significantly different ($P < 0.05$);
[ABC]Mean values within a row with unlike superscripts are significantly different (P < 0.10);
[3]Methods of analysis denoted by ^{G} (Gaussian), ^{n} (Negative Binomial) and ^{p} (Poison).

Table S12. Relative abundance of bacterial phyla in cecal mucosa-associated microbiota of pigs fed control diet (CON), flaxseed meal (FM) and oat hulls (OH)

Phyla present	[1]Diets			[2]SEM	*P-value*[3]
	CON	FM	OH		
---------------Abundant Phylum (>1% of population)---------------					
Bacteroidetes	59.16±5.85[a]	51.22±5.06[ab]	48.89±14.42[b]	4.104	0.008[P]
Firmicutes	36.06±5.26	38.22±7.77	34.90±13.66	5.272	0.257[P]
Proteobacteria	3.05±2.35[b]	9.23±6.26[ab]	13.19±9.37[a]	3.341	<0.001[P]
Unclassified	0.87±1.326	0.514±0.633	2.63±1.11	1.363	0.635[G]
---------------Low abundant Phylum (<1% of population)---------------					
Actinobacteria	0.028±0.03	0.025±0.01	0.021±0.02	0.010	0.743[G]
Cyanobacteria	0.296±0.18	0.269±0.24	0.252±0.13	0.094	0.895[G]
Deferribacteres	0.067±0.14	0.011±0.59	0.033±0.76	0.048	0.882[P]
Elusimicrobia	0.023±0.03	0.016±0.02	0.014±0.02	0.011	0.682[G]
Fibrobacteres	0.014±0.02	0.028±0.02	0.013±0.02	0.010	0.241[G]
Spirochaetes	0.292±0.44[ab]	0.389±0.24[a]	0.093±0.11[b]	0.099	0.024[G]
Synergistetes	0.022±0.01	0.035±0.02	0.024±0.02	0.010	0.286[P]
TM7	0.027±0.02	0.016±0.01	0.013±0.01	0.009	0.202[G]

[1]CON, Control diet; FM, Flaxseed meal-containing diet; OH, Oat hulls-containing diet;
[2]SEM, standard error of means;
[†]Least square mean values (n = 8 replicates per treatment);
[abc]Mean values within a row with unlike superscripts are significantly different (*P* < 0.05);
[ABC]Mean values within a row with unlike superscripts are significantly different (P < 0.10);
[3]Methods of analysis denoted by [G] (Gaussian), [n] (Negative Binomial) and [p] (Poison).

Table S13. Phylogenetic composition of bacterial genus from illumina-sequencing of 16S rRNA sequences in cecal digesta of pigs fed experimental diets

	Diets[1]			SEM[2]	*P-value*[3]
Order (O), Family (F), Genus	CON	FM	OH		
……………………………Actinobacteria (P)………..…………………………					
Bifidobaterium	2.45	0.809	0.497	0.811	0.115^{NB}
Unclassified Coriobacteriaceae (F)	0.0511^{b}	0.164^{a}	0.0875^{b}	0.021	<0.001^{G}
……………….………..…...……Bacteroidetes (P)………..………………………………………					
Unclassified Bacteroidales (O)	4.03^{AB}	2.77^{B}	5.49^{A}	0.873	0.066^{P}
Bacteroides	0.0704^{b}	0.736^{a}	0.159^{b}	0.196	0.006^{G}
Parabacteroides	0.109	0.183	0.122	0.046	0.245^{G}
Prevotella	30.43^{b}	26.73^{b}	38.37^{a}	2.264	0.002^{P}
CF231	1.53^{A}	1.43^{AB}	1.06^{B}	0.192	0.070^{G}
………………………………..…..…Firmicutes (P)………..……………..……………………..					
Lactobacillus	6.41^{b}	16.13^{a}	12.87^{ab}	2.977	<0.001^{P}
Streptococcus	8.29^{a}	5.18^{b}	7.03^{ab}	0.816	0.004^{G}
Unclassified Clostridiales (O)	3.51	3.10	3.14	0.858	0.875^{P}
Unclassified Clostridiaceae (F)	0.210	0.0149	0.159	0.249	0.117^{NB}
Clostridium	0.389^{a}	0.139^{b}	0.214^{ab}	0.077	0.012^{G}
Unclassified Lachnospiraceae (F)	0.572^{a}	0.425^{ab}	0.309^{b}	0.109	0.078^{G}
Blautia	1.263^{AB}	1.061^{B}	1.934^{A}	0.397	0.094^{G}
Coprococcus	0.453^{b}	0.351^{b}	0.824^{a}	0.086	<0.001^{G}
Dorea	0.347^{b}	0.298^{b}	0.491^{a}	0.051	0.004^{G}
Roseburia	0.950^{a}	0.348^{b}	0.458^{b}	0.169	0.004^{G}
Shuttleworthia	0.0364	0.0522	0.0094	0.022	0.152^{P}
Unclassified Ruminococcaceae (F)	3.64	2.99	3.70	0.664	0.498^{G}
Ruminococcus	1.01	0.685	1.03	0.035	0.078^{NB}
Faecalibacterium	0.277	0.325	0.430	0.086	0.219^{G}
Oscillospira	0.496	0.647	0.552	0.186	0.718^{P}
Peptococcus	0.0948	0.0802	0.1078	0.024	0.527^{G}

[1]CON, Control diet; FM, Flaxseed meal-containing diet; OH, Oat hulls-containing diet;
[2]SEM, standard error of means;
†Least square mean values (n = 8 replicates per treatment).
[abc]Mean values within a row with unlike superscripts are significantly different ($P < 0.05$).
[ABC]Mean values within a row with unlike superscripts are significantly different ($P < 0.10$).
[3]Methods of analysis denoted by G (Gaussian), [NB] (Negative Binomial) and [P] (Poison).

Table S13. Continued

	Diets[1]			SEM[2]	*P-value*[3]
Order (O), Family (F), genus	CON	FM	OH		
……………………………….…..….Firmicutes (P)………...……………..………………….					
Unclassified Veillonellaceae	1.21[AB]	1.74[A]	0.658[B]	0.445	0.075[G]
Acidaminococcus	0.426[AB]	0.698[A]	0.148[B]	0.240	0.098[G]
Anaerovibrio	0.910	1.22	0.942	0.319	0.569[G]
Mitsuokella	0.0986	0.0655	0.102	0.032	0.477[G]
Megasphaera	18.85	19.64	16.04	4.035	0.650[P]
Phascolarctobacterium	1.33[b]	3.69[a]	2.70[ab]	0.628	0.011[P]
Unclassified Erysipelotrichaceae (F)	0.00765[b]	0.00953[b]	0.0203[a]	0.007	0.019[G]
Bulleidia	0.0421	0.0407	0.0328	0.016	0.815[G]
Catenibacterium	0.0282	0.0093	0.0250	0.010	0.134[G]
Eubacterium	0.0126[B]	0.0186[B]	0.0453[A]	0.013	0.053[G]
Sharpea	0.0405	0.0283	0.0313	0.024	0.872[G]
………………………..………………..Proteobacteria (P)……...…..………..……………………..					
Unclassified Proteobacteria	0.777[b]	1.831[a]	1.109[ab]	0.364	0.026[NB]
Campylobacter	0.8139	1.6408	1.2250	0.651	0.460[P]
Succinivibrio	0.0951	0.1580	0.0797	0.056	0.352[G]
Sutterella	0.0155	0.0298	0.0266	0.008	0.186[G]

[1]CON, Control diet; FM, Flaxseed meal-containing diet; OH, Oat hulls-containing diet;
[2]SEM, standard error of means;
[†]Least square mean values (n = 8 replicates per treatment).
[abc]Mean values within a row with unlike superscripts are significantly different ($P < 0.05$).
[ABC]Mean values within a row with unlike superscripts are significantly different ($P < 0.10$).
[3]Methods of analysis denoted by G (Gaussian), [NB] (Negative Binomial) and [P] (Poison).

Table S14. Phylogenetic composition of bacterial genus from illumina-sequencing of 16S rRNA sequences in cecal tissue of pigs fed experimental diets

Phylum, Order, Family, genus	Diets[1]			SEM[2]	*P-value*[3]
	CON	FM	OH		
………………………………Actinobacteria (P)………………………………					
Unclassified Coriobacteriaceae	0.0294	0.0248	0.0204	0.010	0.668^{G}
………………………………Bacteroidetes (P)………………………………					
Unclassified Bacteroidales (O)	1.83	1.92	1.40	0.426	0.437^{G}
Bacteroides	0.0865^{B}	0.938^{A}	0.187^{B}	0.376	0.069^{P}
Parabacteroides	0.171^{b}	0.372^{a}	0.213^{b}	0.060	0.007^{G}
Prevotella	7.79^{AB}	7.12^{B}	10.51^{A}	2.986	0.069^{P}
CF231	1.15	1.02	1.02	0.258	0.839^{P}
YRC22	0.445^{A}	1.111^{A}	0.215^{C}	0.049	0.095^{P}
………………………………Firmicutes (P)………………………………					
Lactobacillus	1.44^{a}	1.52^{a}	0.536^{b}	0.392	0.045^{G}
Streptococcus	1.03	0.618	0.757	0.193	0.118^{G}
Unclassified Clostridiales (O)	1.19	2.51	1.68	0.682	0.172^{G}
Unclassified Clostridiaceae (F)	0.414^{A}	0.239^{B}	0.234^{B}	0.083	0.071^{G}
Clostridium	0.165	0.141	0.121	0.034	0.453^{G}
Unclassified Lachnospiraceae (F)	0.414^{a}	0.239^{b}	0.234^{b}	0.083	0.071^{P}
Blautia	0.3131	0.3665	0.4364	0.253	0.888^{G}
Coprococcus	0.239^{a}	0.121^{b}	0.226^{ab}	0.045	0.029^{G}
Dorea	0.199^{A}	0.102^{B}	0.219^{A}	0.047	0.052^{G}
Lachnospira	0.193^{a}	0.0536^{b}	0.0875^{b}	0.044	0.008^{G}
Roseburia	0.588^{a}	0.219^{c}	0.392^{b}	0.063	<0.001^{G}
Unclassified Ruminococcaceae (F)	4.01	2.98	2.92	0.682	0.210^{P}
Ruminococcus	0.259a	0.135b	0.193b	0.032	0.008^{G}
Faecalibacterium	0.814^{a}	0.380^{ab}	0.334^{b}	0.187	0.032^{G}
Oscillospira	1.05^{a}	1.12^{a}	0.462^{b}	0.166	0.027^{G}
Unclassified Peptostreptococcaceae (F)	0.0147	0.0078	0.0124	0.001	0.342^{P}
Peptococcus	0.0246	0.0185	0.0146	0.007	0.413^{NB}

[1]CON, Control diet; FM, Flaxseed meal-containing diet; OH, Oat hulls-containing diet;
[2]SEM, standard error of means;
[†]Least square mean values (n = 8 replicates per treatment).
[abc]Mean values within a row with unlike superscripts are significantly different ($P < 0.05$).
[ABC]Mean values within a row with unlike superscripts are significantly different ($P < 0.10$).
[3]Methods of analysis denoted by G (Gaussian), [NB] (Negative Binomial) and [P] (Poison).

Table S14. Continued

Phylum, Order, Family, genus	Diets[1]			SEM[2]	*P-value*[3]
	CON	FM	OH		
……………Firmicutes (P)……………					
Unclassified Veillonellaceae (F)	3.37a	1.17ab	1.08c	1.051	0.010^{P}
Acidaminococcus	0.733b	3.14a	0.466b	1.190	0.001^{P}
Anaerovibrio	1.98^{b}	2.53^{ab}	5.21^{a}	1.069	0.005^{NB}
Mitsuokella	0.2705	0.1454	0.2411	0.063	0.126^{NB}
Megasphaera	13.40^{a}	10.90ab	6.01b	1.956	0.003^{NB}
Phascolarctobacterium	4.2594	4.7350	4.6488	0.649	0.741^{G}
Veillonella	0.0142^{b}	0.0765^{a}	0.0034^{b}	0.022	0.008^{G}
Unclassified Mogibacteriaceae (F)	3.82^{a}	3.58^{ab}	1.29^{c}	2.137	0.025^{P}
Unclassified Erysipelotrichaceae (F)	0.0761	0.0727	0.1020	0.033	0.634^{G}
……………....Proteobacteria (P)……………					
Unclassified Proteobacteria	0.0755	0.302	0.330	0.156	0.341^{G}
Campylobacter	1.02^{b}	4.35^{ab}	5.28^{a}	2.401	0.029^{NB}
Unclassified Succinivibrionaceae (F)	0.0647	0.318	0.221	0.148	0.216^{G}
Succinivibrio	0.070^{b}	0.101^{ab}	0.164^{a}	0.033	0.033^{G}
Sutterella	1.22^{b}	1.75^{ab}	5.36^{a}	3.084	<0.001^{P}
Unclassified Desulfovibrionaceae (F)	0.034^{B}	0.035^{B}	0.083^{A}	0.053	0.577^{G}
Desulfovibrio	0.131	0.133	0.122	0.050	0.972^{G}
Unclassified Enterobacteraceae (F)	0.110	0.375	0.520	0.268	0.336^{G}
Unclassified Helicobacteraceae (F)	0.016^{B}	1.59^{A}	0.415^{B}	0.897	0.097^{P}
Flexispira	0.069^{B}	0.213^{A}	0.112^{B}	0.080	0.092^{G}
Helicobacter	0.099	0.0639	0.4830	0.326	0.379^{G}

[1]CON, Control diet; FM, Flaxseed meal-containing diet; OH, Oat hulls-containing diet;
[2]SEM, standard error of means;
[†]Least square mean values (n = 8 replicates per treatment).
[abc]Mean values within a row with unlike superscripts are significantly different (*P* < 0.05).
[ABC]Mean values within a row with unlike superscripts are significantly different (*P* < 0.10).
[3]Methods of analysis denoted by G (Gaussian), [NB] (Negative Binomial) and [P] (Poison).

Table S15. Correlations coefficients between selected bacterial taxa[a] and short chain fatty acids (SCFA), bile acids (BA) and neutral sterols (NS) in ileal digesta

Taxa	Variable[1]	Rho	*P*-value
YRC22	Cholesterol	0.3599	0.036
Lactobacillus	CA-CDCA	0.4273	0.023
	IDCA	-0.6441	<0.001
Streptococcus	Fat digestibility	0.4170	0.017
Clostridiales (O)	Propionate	0.4656	0.046
	LCA	0.4752	0.042
	DCA	0.5105	0.035
	IDCA	0.3875	0.007
	UDCA	0.6003	0.004
Clostridiaceae (F)	LCA	0.3806	0.042
Erysipelotrichaceae (F)	Cholesterol	0.3431	0.046
	Triglycerides	0.6132	0.003
Mitsuokella	Triglycerides	0.5700	0.008
Coriobacteriaceae (F)	Propionate	0.5607	0.003
Veillonellaceae (F)	Acetate	0.5341	0.002
	LCA	-0.3456	0.041
	IDCA	-0.4135	0.011
Acidaminococcus	CA-CDCA	-0.4640	0.033
	Triglycerides	0.5066	0.028
Phascolarctobacterium	Propionate	0.4327	0.015

[a]Bacterial taxa with relative abundance ≥0.05% of the community were used for the correlation analysis. Only significant correlations are presented in the Table. [1]CA = Cholic acid; CDCA = Chenodeoxycholic acid; DCA = Deoxycholic acid; IDCA = Isodeoxycholic acid; LCA = Lithocholic acid; UDCA = Ursodeoxycholic acid..

Table S16. Correlations coefficients between selected bacterial taxa[a] and short chain fatty acids (SCFA), and bile acids (BA) in ileal mucosa

Taxa	Variable[1]	r	*P*-value
Lactobacillus	Propionate	0.7307	<0.001
	LCA	0.5252	0.043
	DCA	0.6176	0.008
	IDCA	0.6402	0.005
Streptococcus	Acetate	0.7306	<0.001
	DCA	0.4729	0.004
Veillonella	Propionate	0.8992	<0.001
	Butyrate	0.4581	0.044
	Valerate	0.5252	0.027

[a]Bacterial taxa with relative abundance ≥0.05% of the community were used for the correlation analysis. Only significant correlations are presented in the Table. [1]DCA = Deoxycholic acid; IDCA = Isodeoxycholic acid; LCA = Lithocholic acid.

Table S17. Correlations coefficient between selected bacterial taxa and short chain fatty acids (SCFA), bile acids (BA) and neutral sterols (NS) in cecal digesta

Taxa	Variable[1]	r	*P*-value
Bacteroidales (O)	Butyrate	-0.3998	0.025
	Valerate	-0.4167	0.015
Bacteroides	UDCA	0.4356	0.022
Prevotella	Propionate	-0.3714	0.042
	Butyrate	-0.3908	0.036
	Valerate	-0.4616	0.024
	CA-CDCA	0.5745	0.011
	UDCA	0.5745	0.002
Lactobacillus	Coprostanol	0.5378	0.007
	DCA	0.6970	0.041
	UDCA	0.5666	0.039
Streptococcus	Acetate	-0.4622	0.045
	Valerate	-0.3419	0.022
	Fat digestibility	0.6188	0.004
	DCA	-0.5795	0.008
	UDCA	-0.5340	0.037
	Triglycerides	-0.5667	0.009
Lachnospiraceae (F)	CA-CDCA	-0.4578	0.041
	Coprostanol	-0.5132	0.022
Clostridium	Acetate	-0.4714	0.035
	Butyrate	-0.3530	0.043
	DCA	-0.5007	0.038
	UDCA	-0.4942	0.026
	Triglycerides	-0.4219	0.019
Coprococcus	Propionate	-0.5650	0.007
	Butyrate	-0.5007	0.013
	Valerate	-0.4789	0.043
	CA-CDCA	0.5822	0.004
	IDCA	0.6012	<0.001

Table S17. Continued

Taxa	Variable	r	*P*-value
Dorea	Butyrate	-0.4504	0.034
	Valerate	-0.4861	0.046
	Fat digestibility	0.3802	0.025
	CA-CDCA	0.4904	0.016
	Coprostanol	0.4037	0.024
	IDCA	0.4904	0.044
	Triglycerides	-0.3708	0.039
Roseburia	Acetate	-0.5218	0.004
	CA-CDCA	-0.4552	0.028
	Coprostanol	-0.5245	0.032
	DCA	-0.5645	0.003
	UDCA	-0.5833	0.008
	IDCA	-0.4552	0.043
	Triglycerides	-0.4846	0.019
Coriobacteriaceae (F)	Acetate	0.6095	0.004
	Butyrate	0.3965	0.048
	Valerate	-0.4255	0.029
	DCA	0.4422	0.043
	IDCA	0.5541	0.002
	Triglycerides	0.7751	<0.001
	Coprostanol	0.4950	0.035
Acidaminococcus	Butyrate	0.5068	0.047
	Valerate	0.6991	<0.001
	Fat digestibility	-0.6062	0.004
Phascolarctobacterium	Coprostanol	0.3608	0.047
	DCA	0.4086	0.042
	UDCA	0.3899	0.015
Eubacterium	Coprostanol	0.4207	0.049

[a]Bacterial taxa with relative abundance ≥0.05% of the community were used for the correlation analysis. Only significant correlations are presented in the Table. CA = Cholic acid; CDCA = Chenodeoxycholic acid; DCA = Deoxycholic acid; IDCA = Isodeoxycholic acid; LCA = Lithocholic acid; UDCA = Ursodeoxycholic acid.

Table S18. Correlations coefficient between selected taxa[a] and volatile fatty acids, bile acids and neutral sterols (NS) in cecal tissue

Taxa	Variable	r	*P*-value
Bacteroides	DCA	0.3666	0.069
	UDCA	0.4050	0.042
Provotella	Cholesterol	-0.6174	0.008
	Triglycerides	-0.5827	0.005
	Acetate	0.4456	0.018
	CA-CDCA	-0.3948	0.037
	Coprostanol	-0.6315	0.006
Parabacteroides	Acetate	0.3706	0.045
	Butyrate	0.4061	0.011
	Coprostanol	0.4124	0.044
	DCA	0.4562	0.016
	UDCA	0.5190	0.007
YRC22	Propionate	0.3656	0.065
	LCA	0.3431	0.061
Clostridiaceae (F)	CA-CDCA	-0.4104	0.038
	Coprostanol	-0.6296	0.004
	DCA	-0.3893	0.057
Lachnospira	Butyrate	0.5034	0.006
	Fat digestibility	0.5512	0.003
	Coprostanol	-0.5478	0.002
	LCA	-0.3703	0.068
	DCA	-0.5556	0.005
	UDCA	-0.5801	0.011
Coprococcus	Propionate	-0.3701	0.055
	Butyrate	-0.3557	0.079
	Fat digestibility	0.6607	<0.001

Table S18. Continued

Taxa	Variable	r	*P*-value
Dorea	Triglycerides	0.3889	0.044
Megasphaera	LCA	0.4200	0.015
Roseburia	Valerate	-0.4192	0.046
Veillonella	Fat digestibility	-0.3491	0.056
	CA-CDCA	-0.4002	0.088
	Coprostanol	-0.3649	0.072
	LCA	0.5264	0.005
	DCA	0.4311	0.014
	UDCA	0.5726	0.002
Mitsuokella	Acetate	-0.3522	0.091
	UDCA	-0.3762	0.055
Succinivibrionaceae	Acetate	0.3968	0.041
	Coprostanol	0.3541	0.073
	LCA	0.4244	0.014
	DCA	0.5464	0.005
	UDCA	0.4657	0.029
Veillonellaceae	Acetate	-0.5216	0.008
	DCA	-0.4513	0.047
	UDCA	-0.3881	0.062
Acidaminococcus	Butyrate	0.4308	0.037
	Valerate	0.6503	<0.001
	Cholesterol	0.3833	0.053
	Fat digestibility	-0.6984	<0.001
Anaerovibrio	Acetate	0.4888	0.046
	CA-CDCA	0.4791	0.023
	Coprostanol	0.6271	0.004
Shutella	Acetate	0.4422	0.039
	CA-CDCA	0.4553	0.016
	LCA	0.3933	0.031
	DCA	0.3862	0.025
Campylobacter	Acetate	0.5093	0.030
	CA-CDCA	0.4209	0.044
	Coprostanol	0.6016	0.007
Succinivibrio	Acetate	0.4423	0.014
	Coprostanol	0.4318	0.034
Flexispira	Valerate	0.4448	0.010
	Fat digestibility	-0.4661	0.057
Helicobacteraceae	Valerate	0.5951	0.008
	Cholesterol	0.3774	0.063
	Fat digestibility	-0.7192	<0.001

[a]Bacterial taxa with relative abundance ≥0.05% of the community were used for the correlation analysis. Only significant correlations are presented in the Table. CA = Cholic acid; CDCA = Chenodeoxycholic acid; DCA = Deoxycholic acid; IDCA = Isodeoxycholic acid; LCA = Lithocholic acid; UDCA = Ursodeoxycholic acid.
